# Water-Dispersible Three-Dimensional LC-Nanoresonators

**DOI:** 10.1371/journal.pone.0105474

**Published:** 2014-08-25

**Authors:** Vito Clericò, Luca Masini, Adriano Boni, Sandro Meucci, Marco Cecchini, Fabio A. Recchia, Alessandro Tredicucci, Angelo Bifone

**Affiliations:** 1 Institute of Life Sciences, Scuola Superiore Sant'Anna, Pisa, Italy; 2 Center for Nanotechnology Innovation @NEST, Istituto Italiano di Tecnologia, Pisa, Italy; 3 NEST, CNR-Istituto Nanoscienze and Scuola Normale Superiore, Pisa, Italy; 4 Department of Physiology, Temple University School of Medicine, Philadelphia, Pennsylvania, United States of America; University of California, Merced, United States of America

## Abstract

Nanolithography techniques enable the fabrication of complex nanodevices that can be used for biosensing purposes. However, these devices are normally supported by a substrate and their use is limited to in vitro applications. Following a top-down procedure, we designed and fabricated composite inductance-capacitance (LC) nanoresonators that can be detached from their substrate and dispersed in water. The multimaterial composition of these resonators makes it possible to differentially functionalize different parts of the device to obtain stable aqueous suspensions and multi-sensing capabilities. For the first time, we demonstrate detection of these devices in an aqueous environment, and we show that they can be sensitized to their local environment and to chemical binding of specific molecular moieties. The possibility to optically probe the nanoresonator resonance in liquid dispersions paves the way to a variety of new applications, including injection into living organisms for in vivo sensing and imaging.

## Introduction

Plasmonic and metamaterial devices allow the manipulation and control of electromagnetic waves on a scale smaller than the wavelength of the radiation used to probe their optical response [1] [2]. Thanks to the strong localization and enhancement of the electromagnetic fields they can be used as sensors [3] of the interactions with their local environment with high sensitivity and spatial resolution. In the last decade, we have witnessed a growing development of plasmonic devices as imaging agents [4] for sensing applications in biology [5] [6], food science [7] environmental sciences [8] and medical diagnostics [9]. Chemical synthesis is an established technique to produce metal nanoparticles whose surfaces can be functionalized to obtain stable dispersions in liquids, and to selectively detect molecular interactions [10]. This bottom-up approach, however, presents some important limitations. Firstly, the optical properties of these nanoparticles are determined by the intrinsic characteristics of the material they are made of, and can be tuned within a very limited range of resonance frequencies [11]. Moreover, the repertoire of structures that can be obtained by chemical synthesis is limited, thus constraining the possibility to tailor and fine-tune their properties by changing shape and size. Alternatively, top-down nanofabrication approaches offer great control of these last two parameters. For example, a procedure to fabricate polymer nanoparticles was presented in [12], though here no tailoring of the electromagnetic response was pursued, but rather the implementation of carriers for drug delivery. Other authors [13] presented top-down nanofabrication techniques to generate asymmetric metallic particles for specific applications (i.e. photothermal therapy [14]), but this approach is restricted to pyramidal shapes and has limited capabilities of fine-tuning the optical response. Recently, nanocolloidal suspensions of low-symmetry nanostructures made of different functional materials have been demonstrated, overcoming some of these limitations [15]. Metamaterials possess properties that depend strictly on the geometry of the device rather than their composition [16] and can be designed almost at will to have an optical resonant response in widely different parts of the electromagnetic spectrum, from the visible [17] and near Infrared [18] [19] down to the Terahertz [20] [21] and Gigahertz [22]. Modern nanolithography techniques make it possible to obtain composite nanodevices with complex shapes and accurately controllable properties. The main drawback of these structures, however, is that they are normally anchored on a substrate and cannot be suspended in a solution, with limitations to the range of possible applications. By way of example, these devices may be used for “in vitro” bioassays, but they cannot be injected into a living organism and tracked “in vivo”. Among metamaterials, those based on LC circuit elements are particularly interesting. Indeed their working principle can be described as the resonance of a nanoscopic circuit, whose electromagnetic response can be tailored and finely tuned by design. In their most common configuration, these devices are based on a split-ring geometry, consisting of arrays of metallic (typically Au) open rings [23]. In this case, the gap can be seen as a capacitor (C) and the metallic loop as an inductive (L) element. The values of C and L depend on the geometric parameters [24]; therefore the resonance (w = 1/√LC) can be easily tuned scaling the dimensions of the object [25]. However, split rings are intrinsically two-dimensional, and cannot be easily detached from their substrate and suspended as free-standing devices. Here, we have used nanolithography to produce composite, three-dimensional LC nanoresonators [26] that can be detached and dispersed in a liquid medium. A multistrate design comprising three different materials enables facile multifunctionalization of the resonators, and the formation of stable aqueous supensions while maintaining sensing capabilities. By way of example, we designed and fabricated nanoresonators with an optical resonance in the near-infrared region, where absorption by water and other components in biological tissues (Hb, HbO2, Melanin, Protein) is low [27], thus making them suitable for detection in living cells and tissues. We demonstrated optical detection of the resonators in an aqueous suspension, and sensitivity of their optical response to the microenvironment.

These features significantly extend the range of applications of nanoresonators, e.g. as diagnostic media, for sensing physiological and biochemical events in microfluidic assays, or to impregnate porous materials.

## Results and Discussion

### LC nanoresonator design and simulations

**Figure 1:**
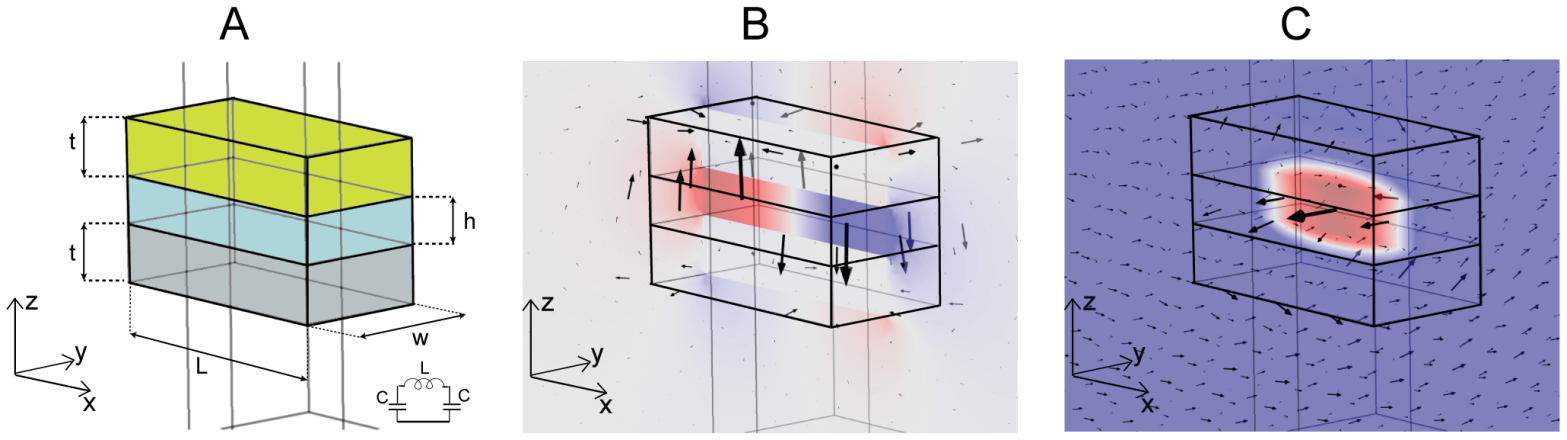
Design and LC behaviour. (A) Design of a three-dimensional nanoresonator and its schematization as nanocircuit (B–C) Simulations with Comsol 4.2: z-component of the electic and norm of the magnetic fields (colour plot) demostrate the LC-behaviour at NIR wavelengths. The arrows represent the direction and sign of electric and magnetic field.

Figure 1A displays a schematic view of the device we have fabricated, which has a three dimensional, wafer structure consisting of three layers of aluminium (bottom layer), aluminium oxide (middle layer) and gold (top layer). The choice of two different metals for the two facets plays a crucial role in the fabrication procedure. In fact this allows a selective etching of the bottom layer without affecting the top metal layer, thus making it possible to physically detach the resonators from the substrate. We fabricated various arrays of these nanoresonators, with thicknesses of 50 nm for both metal layers and 40 nm for the dielectric layer, and lateral sizes (x,y) between (100,200) nm and (125,250) nm. This device can be described as a nanoscopic circuit (Figure 1A) excited by a harmonic magnetic field with a component normal to the plane (x,z). In this case two antiparallel currents in the metals (a loop current) are generated, and this loop current determines an inductive regime, while the accumulation of opposite charges in the extremities of the upper and lower metal slabs creates a capacitive coupling in those areas. This LC behaviour is shown in Figure 1B–C, where we simulated the z component of the electric field (B) and the magnetic field norm (C) with a finite-element software. The three components of the electric field are represented by arrows, showing that the main contribution is due to the z-component. The confinement of the electric field at the extremities of the two metal slabs confirms their functioning as capacitive elements. The arrows indicate also that the main contribution of the magnetic field is along the y-direction, perpendicular to the ‘wires’ and in this case the confinement in the central resonator part represents the inductive element. The electric and magnetic fields, however, are not entirely confined within the device. Hence, the resulting LC resonance, beyond the dependence on size and shape, is influenced by the external dielectric constant and magnetic permittivity, and can be used as a way to probe the local environment and chemical binding at the surface of the nanodevice.

### LC nanoresonators on a substrate

**Figure 2:**
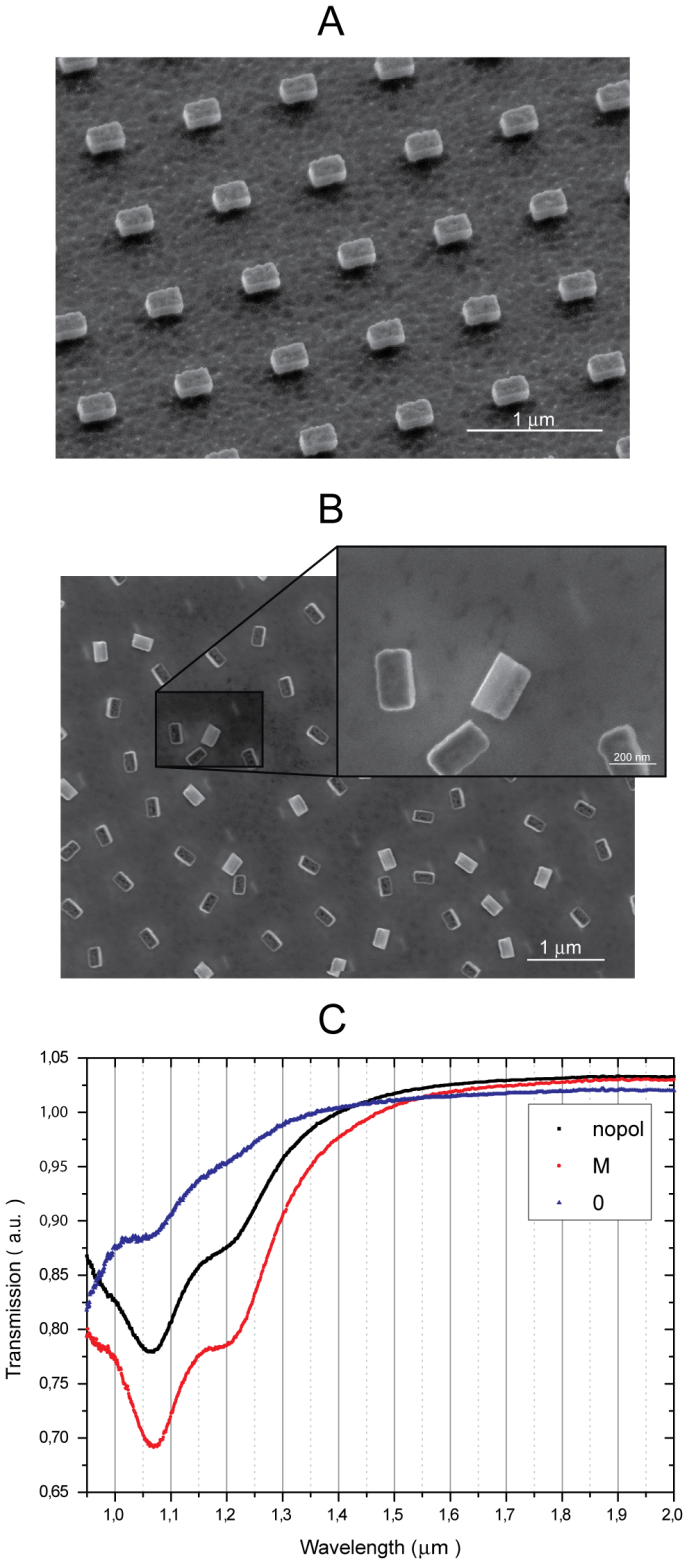
Nanoresonators and their characterization. (A) SEM-image of the nanoresonators disposed in array (B) SEM-image of nanoresonators pulled off the substrate and randomly oriented (C) Transmission spectrum of nanoresonators disposed in array for different polarizations.

Figure 2A–B shows the nanoresonators (230 nm×115 nm lateral sizes) disposed in an array (A) or detached (random orientation) (B) on a Gallium Arsenide substrate. In the zoomed view of Figure 2B, the three-layer structure is apparent in the capsized nanoresonator. To study the resonance properties of the nanoresonators disposed in a two-dimensional ordered array, we used a commercial Fourier-transform (FTIR) Nexus spectrometer equipped with a white light lamp and a PbSe detector, with an incident beam perpendicular to the sample surface and in different polarization conditions: unpolarizated, or linearly polarized with the magnetic field perpendicular or parallel to the long side of the slab. In the transmission spectrum (Figure 2C) the resonance is clearly visible around 1.05 µm. Note that below 1 µm wavelength the gallium arsenide substrate starts to absorb the radiation and for this reason we could not measure the resonance shape at shorter wavelengths. As expected, the strongest coupling is with the light polarized with the magnetic field perpendicular to the ‘wire’ (M-polarization). A small coupling between light and nanoresonator is also present in the complementary polarization (0-polarization), due to scattering. A second small dip, red-shifted with respect to the main one, appears in both cases. Notably, the resonance contrast with unpolarized (nopol) light is between 20–30%, thus suggesting the possibility to detect the resonance even when the nanoresonators are randomly oriented, as in a liquid suspension. A preliminary estimate of the level of coupling in a situation of isotropically oriented nanoresonators can be derived from the resonance of pulled-off nanoresonators on a Gallium Arsenide substrate. In this case we measured a contrast around 3–4%, with the same density (1 per µm^2^) of nanoresonators disposed in array (Text S1 and Figure S1). It is also important to highlight that the distance among the nanoresonators does not affect the position of the resonance (Text S2 and Figure S2D). This fact confirms that the resonant mode is determined only by the size and shape of the individual nanoresonators, and is not due to a photonic crystal structure.

### LC nanosensors in a microfluidic chip

**Figure 3:**
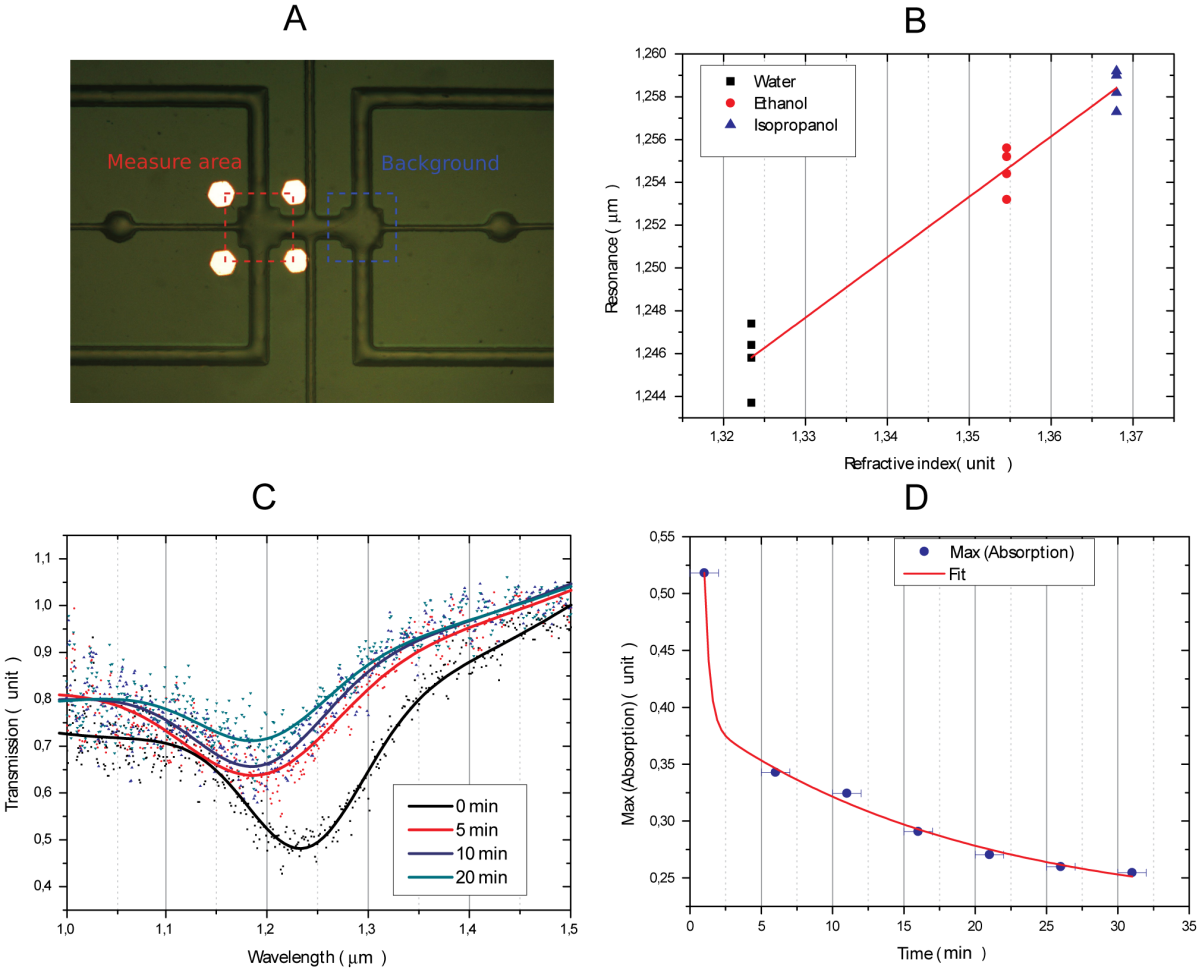
Nanoresonators in a microfluidic chip. (A) Microfluidic chip with gold markers (B) Shift of the resonance for three liquids with different refractive indexes injected into the chamber (C) Transmission spectra resolved in time during cysteamine-binding (D) Study of Cysteamine-binding kinetics. The error bar is taken as half the acquisition time of every single measurement.

To demonstrate the use of these devices as nanosensors, we fabricated an array of nanostructures of 250*125 nm lateral size on glass (instead of Gallium Arsenide), and we controlled their chemical environment with the aid of a microfluidic device (Figure 3A). The microfluidic chip had two microchambers (one for the sample and the other to measure the background signal) and reference markers to control the spot size and to align the beam focus on the array of nanoresonators. Before and after each measurement, the microchamber was cleaned with DI water and dried for 15 minutes by exposure to an external lamp. In Figure 3B the shift of the resonance is reported as a function of the refractive index of the different liquids (water, ethanol, isopropanol) that were injected into the microfluidic chip [28] [29] [30]. The four data-sets were acquired at room temperature in four separate experimental sessions and on two different days to test the repeatability of the measurement. The time duration of each measurement was 30 minutes (for the sample and the background). The bulk sensitivity S, that is defined as the spectral shift Δλ (in nanometers) originated by a certain refractive index change Δn of the external environment, was calculated from the slope of the linear fit, resulting in S = 280 nm per refractive index unit (RIU). The performance of the device is determined by its figure of merit (FOM), which can be calculated as the sensitivity (S) divided by the full width half maximum (FWHM) of the resonance. We found a FOM of almost 6, lower [31] or comparable [32] to other similar plasmonic biosensors. For this sample we used a dry etching step in the nanofabrication process, which is necessary to completely define the structure. While this process may have resulted in a slight deterioration of the edge of the structure, resulting in a reduction of the figure of merit, it also brings some advantages, including the possibility to functionalize different layers of the nanoresonators and, most importantly, to pull off and disperse the nanoresonators in aqueous media, as described in the next section.

As well as by the dielectric properties of the external environment, the resonance of these nanodevices is influenced by chemical binding of molecules to one of the metal facets, enabling the detection of specific molecular interactions or the kinetic study of chemical binding. We measured the transmission spectra of the nanoresonators at different time-points while a solution (1∶1000) of cysteamine and water was flowing through the microfluidic chip. In Figure 3C we report a few exemplary measurements. Each spectrum acquisition time lasted 2 minutes, while the interval between two subsequent acquisitions was 5 minutes. Cysteamine is known to bind to the gold layer with its thiol groups [33]; in a circuit model, it acts as an external impedance that causes a damping of the resonance [34]. This behaviour is apparent in Figure 3C, where damping of the resonance and a blue shift are visible in the interval 0 to 30 minutes. The absorption was calculated as 1-min(Transmission) and showed a time-dependence similar to that reported in [35], as shown in Figure 3D.

### LC nanosensors in suspension

The possibility to suspend our nanoresonators in a liquid medium is critical for many applications, especially in the biological field. To this end, we applied selective wet etching (see Methods for details) to pull the nanoresonators off the substrate, as shown in Figure 2B.

**Figure 4:**
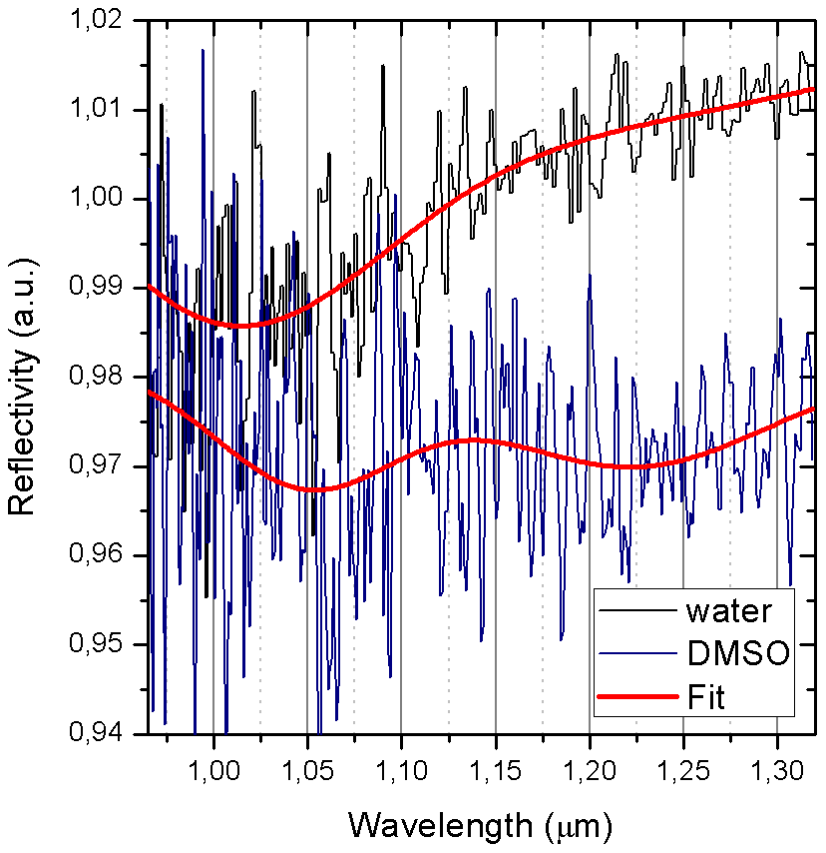
Sensing with suspended nanoresonators. Spectra of nanoresonators in water and DMSO.

After etching, most of the nanoresonators remained on the substrate due to Van der Waals adhesion forces. Their dispersal in the liquid of choice was achieved by robust sonication in an ultrasonic bath. To obtain a stable suspension in water (or other liquids), we first dispersed the nanoresonators in DMF (Dimethylformamide), where they were coated with PEGylated 3-aminopropyltriethoxysilane (APTES), an alkalosilane that helps prevent aggregation and precipitation [36]. The APTES-PEG molecule reacts with the oxydryl moieties that are present on the aluminium and aluminium oxide surfaces, resulting in a Si-O-Al bond. Importantly, the APTES-PEG does not bind to the gold layer, thus making it possible to functionalize this layer with different moieties for sensing purposes. After removal of the solvent under vacuum, the coated nanostructures were dispersed in water by sonication and purified by dialysis. To demonstrate the feasibility of detection in the liquid phase, nanoresonators were suspended in 1 µl of water or DMSO (Dimethyl sulfoxide), and the samples were measured in small SU8-on-glass chambers of 5*5 mm lateral size and 0.04 mm depth. From Inductively Coupled Plasma-Mass Spectrometry technique (ICP-MS, Agilent Technologies Inc., 7700 series, Usa) we estimated that a number between 2.7 * 10^6^ and 0.83 * 10^7^ of nanoresonators were suspended. Reflectance measurements were performed using a commercial insert for fixed 10° incidence angle (Pyke 10 Spec). In Figure 4 we report the extinction spectra of the nanoresonators dispersed in two different solvents, water and DMSO, respectively, characterized by different refractive indexes. In both cases, we found a blue shift of the resonance with respect to the measurement in the array, which was likely dominated by the high refractive index of the Gallium Arsenide substrate. The ratio between the shift of water and DMSO is 4.25, in good agreement with the value (4.43) estimated from simulations by FEM analysis (Comsol Multiphysics 4.2). The contrast of the resonance is about 1%, a factor of 20 lower than for the array, consistent with the random orientation of the nanoresonators in the liquid suspension, and with the damping of the resonance due to the molecular binding of APTES. Additionally, the Fabry-Perot interference between the two glasses of the chamber enclosure is superimposed to our signal, and makes it difficult to estimate the SNR. However, we can make a conservative estimate of the Contrast to Noise ratio (CNR), i.e. the ratio between the absorption change induced by the solvent at 1.25 nm wavelength and the apparent noise (which includes the FP oscillations). We obtain a CNR≈4–5, sufficient to appreciate the shift of the resonance peak with changing medium, thus demonstrating the potential use of these devices as nanosensors in aqueous environment.

## Discussion

LC nanocircuits can be tuned over a very wide range of resonance frequencies, and offer an unprecedented opportunity for the development of flexible sensors that can operate in very different regions of the electromagnetic spectrum. However, the intrinsic 2-dimensional nature of these metamaterials has so far limited their use, particularly in the biological field. Here, we have proposed a method to produce a stable aqueous suspension of three-dimensional nanoresonators that can be used as a contrast agent or as biosensors for optical imaging applications. Our procedure is conceptually different from those used to produce imaging agents, which are typically made from chemical synthesis. Indeed, here we have taken a top-down approach, starting with *in silico* modelling to tailor the desired optical response of the nanodevice, and the subsequent nanofabrication using nanolithography. Several elements of novelty provide enabling steps in this procedure. Firstly, the use of different layered materials makes it possible to detach the nanoresonators from the substrate by selective etching while preserving their structure and resonant properties. Secondly, the coating of our devices with alkalosilanes prevents precipitation in the liquid phase and results in stable colloidal suspension in aqueous media. Importantly, the composite structure enabled us to selectively silanize certain parts of the device, while leaving the gold facet uncoated and available for sensing purposes or further functionalization. This procedure represents a general and flexible method for the fabrication of nanocircuits and their subsequent transfer into a stable suspension independently of their shape, size and design. Finally, their optical response can be defined by design rather than being a mere consequence of their composition, like for plasmonic nanoparticles. To the best of our knowledge, this is the first example of resonating nanocircuits obtained with a top-down approach and suspended in a stable aqueous solution. The sensitivity of the resonance peak position and line-shape to the chemical-physical features of the local environment makes these devices promising for sensing applications. Moreover, the strong localization of the optical signal can be used for the detection of molecular interactions in vivo or in bioassays, for example for the detection of specific proteins or enzymes. The size of our nanoresonators is of the order of 100 nanometers, substantially smaller than the micrometer size of cells, thus making it conceivable to use these devices as intracellular sensors. To this end, the gold facet, free of alkalosilanes, would provide the ideal substrate for the easy functionalization of the device with molecular moieties, like polypeptides, that facilitate cell internalization or the targeting and accumulation in specific tissues. Importantly, we demonstrated detection of our nanoresonators in the aqueous phase, a critical step for their application as bioimaging agents. In the liquid suspension the contrast over the background response was small, but significant, about 1%. One limiting factor for the sensitivity of our measurement was the relatively small number of nanoresonators that we could obtain with our lithography technique (at most 1*10^7^, orders of magnitude lower than the typical number of plasmonic nanoparticles used for similar applications). Consequently, the extinction spectra were measured from a sample of less than a microliter, very small compared to the typical voxel size of many in vivo bioimaging methods. However, alternative nanofabrication techniques, like nanoimprinting [37], could be used to scale up the fabrication of nanoresonators by at least a factor 100. Another important limitation was the anisotropic coupling with the incident radiation that drastically reduces sensitivity when the nanoresonators are randomly oriented, as in a liquid suspension. This is a consequence of the particular structure we have chosen, and a more isotropic design would be desirable. Several isotropic metamaterials have been recently proposed [38], providing a means to improve sensitivity of detection in the liquid phase. For this proof of concept, we have designed nanodevices that resonate in the so-called “biological window”, a spectral region in the near-infrared where absorption by water and hemoglobin [39] is lowest. This region is particularly suited for biomedical applications, and is the region of choice for the development of contrast agents for optical bioimaging. Moreover, these sensors could be functionalized to detect the binding of specific ligands, thus making it possible to use them as detectors of specific molecular moieties. LC nanoresonators can be tuned by design over a wide range of resonance frequencies, down to the terahertz (Text S3 and Figure S3), something that cannot be obtained with plasmonic, chemically-synthesized nanoparticles, thus substantially extending the range of potential applications. By way of example, we could envisage use of these structures for the study of porous materials impregnated with a liquid suspension of nanodevices resonating in a region in which the material is transparent, to maximize contrast. Hence, our nanoresonators may represent a universal scaffold to develop optical probes and sensors for a wide variety of assays in the liquid phase.

## Materials and Methods

### Nanoresonators on GaAs substrate

**Figure 5:**
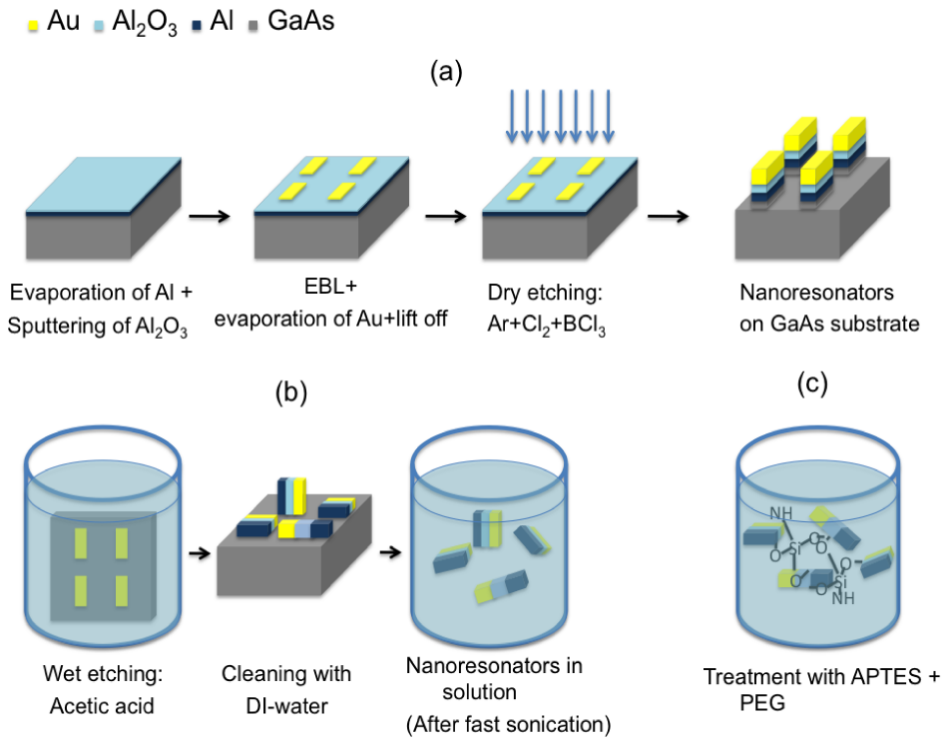
Nanofabrication process and chemical functionalization. (a) top-down approach to define the nanostructure (b) selective wet etching to pull the nanoresonators off the substrate (c) chemical functionalization to suspend the nanoresonators in watery environment.

Our nanofabrication process consists of three conceptually different steps:

- top-down design and lithographic fabrication of the nanostructures on a flat substrate (Figure 5a)
- wet etching to pull the nanoresonators off the substrate (Figure 5b);
- conjugation of the resonators with chemical agents that enable the formation of a stable colloidal suspension in a liquid medium (Figure 5c)

Step (a) involved: Evaporation of Al-film (50 nm); Atomic layer deposition of Al_2_ O_3_ film (40 nm); Electron-beam-lithography; Evaporation of chromium (5 nm), gold (50 nm) and 10 nm of nickel (mask layer); lift-off; Dry-etching with ICP-RIE (gas: BCl_3_,Cl_2_,Ar). At this stage, the nanoresonators were disposed in a regular array, as in Figure 5(a). In step (b), we pulled the nanoresonators off the pedestal of Gallium Arsenide. This was achieved by applying a wet etching technique with acetic acid and hydrogen peroxide. The concentration of the solution was: (C_2_H_4_O_2_:H_2_O:H_2_O_2_) = (20 µm:6 ml:2 ml) and the sample was maintained in solution for 4/5 hours at room temperature (we controlled this step with SEM imaging). The results of this procedure are shown in Figure 5(b). After etching, most of the nanoresonators were capsized along the longer side, but remained on the substrate due to adhesion forces. At the end of this stage the sample was cleaned with DI water and dried. The next step consisted in dispersing the nanostructures in a liquid medium and to control that almost all nanoresonators leave the substrate. This process was facilitated by robust sonication in N,N dimethylformamide (DMF).

In order to disperse the nanostructure in water and prevent aggregation and precipitation, the nanodevices were coated with the alkalosilane APTES ((3-aminipropyl)-trirthoxylane, modified with a PEG chain (MW 550) through the amino group, to improve the stability and the biocompatibility of the suspension. APTES also carries ethoxysilane groups, which are reactive towards hydroxilated surfaces, providing formation of a Si-O-Surface bond (Figure 5(c)). Due to the fact that the gold surface does not present hydroxyl groups, only the aluminium oxide and aluminium surface were bound by APTES, thus leaving the gold face free for additional functionalization. The reaction was performed directly in DMF suspension, upon addition of the PE-modified APTES and acetic acid, which acted as catalyst. After removal of the solvent under vacuum, the functionalized nanostructures were dispersed in water by sonication and purified by dialysis in membrane (cut-off 10 KDa).

### Nanoresonators in a microfluidic chip

For the microfluidic measurements we had to fabricate nanoresonators directly on a glass substrate. The size of the chamber was 250×250×30 µm^3^ and we used a spot size of less than 100 µm, with a homemade optical setup external to the FTIR. The flow of the fluid in the microchannels was controlled by an external pump and it was set to 0.4 µl/min. For nanoresonators, the nanofabrication process is the same of Figure 5a, but we used a glass substrate instead of gallium arsenide and, before the electron beam lithography, we made markers on the glass with optical lithography and an evaporation of gold and nickel. Microchannels were created by soft lithography, pouring PDMS onto an AZ9260 (MicroChem) mold. The air bubbles were removed under vacuum for some minutes and the PDMS baked at 80 °C for 4 hours. We functionalized PDMS and glass surfaces with a low plasma exposure and stitched them together as soon as possible, by aligning the chamber on the markers. Lastly, the microfluidic chip was baked for 4 hours at 80°C. The master mold for the fluidic channels was fabricated spincoating a layer of AZ9260 for 30 s at 1500 rpm on a silicon wafer and placing it on a hotplate at 110°C for 80 s. Then a second layer of AZ9260 was deposited over the first one using the same spincoating parameters and the sample was placed on a hotplate at 115°C for 180 s, obtaining a single photoresist layer 30 µm thick. The channels were fabricated by optical lithography with a Suss MJB4 mask aligner (Suss, MicroTec) exposing the AZ9260 for 113 s at a constant light intensity of 15 mW/cm2. The sample was developed immersing it for 4 min in a solution 1∶3 of AZ 400 K (MicroChem) in water, using deionized water to stop the development. The mold was then placed on a hotplate at 110°C for 2 min and finally exposed to vapours of chlorotrimethylsilane (Sigma-Aldrich) for 15 min in order to facilitate the master-replica detachment.

### SU-8 chamber

We realized an homemade chamber, by lithographycally defining a negative resist (SU-8 2025) on glass. The negative resist (SU-8 2025) was spinned on glass in two steps, the first one was at 500 rpm (ramp-up at 100 rpm/s) for 10 s, the second one at 1900 rpm (ramp-up at 300 rpm/s) for 30 s. The resist was baked at 65 °C for 3′ and at 95°C for 6′. We made an optical lithography in which the exposure time was set at 10″. After a postbake of 1′ at 65°C and 6′ at 95°C, we developed for 5′ in SU-8 developer, stopped in isopropanol for 30″ and baked at 200°C for 15′.

## Conclusion

In conclusion, we have used a top-down approach to fabricate three-dimensional nanoresonators that can be detached from their substrate and suspended in a liquid medium. Crucially, we have exploited the composite structure of these nanoresonators to selectively functionalize certain regions of the device with alkalosilanes, thus obtaining stable colloidal suspensions, while leaving the metallic facets free for sensing purposes, or for further functionalization with other moieties. Importantly, we demonstrated that our nanoresonators maintain sensing capabilities and can be optically detected when suspended in a stable, liquid dispersion. This paves the way to a variety of new applications, including injection into living organisms, adsorption in porous media and use in microfluidic assays.

## Supporting Information

Figure S1 **Characterization of pulled off nanoresonators.** Reflectivity spectrum of pulled off nanoresonators.
(TIF)

Figure S2 **Bone shape nanoresonators.** (A) Design of nanoresonators (B) Simulations of electric (z-component) and magnetic field (norm). The LC- behaviour is clearly visible (C) SEM image of nanoresonators disposed in array (D) Reflectivity of nanoresonators placed at different distances.
(TIF)

Figure S3 **Thz LC nanoresonators.** SEM image of LC nanoresonator for THz range.
(TIF)

Table S1 **Figure 2C** **data.**
(PDF)

Table S2 **Figure 3B** **data.**
(PDF)

Table S3 **Figure 3C** **data.**
(PDF)

Table S4 **Figure 3D** **data.**
(PDF)

Table S5 **Figure 4** **data.**
(PDF)

Table S6 **Figure S1 data.**
(PDF)

Table S7 **Figure S2D data.**
(PDF)

Text S1 **Contrast of randomly oriented nanoresonators.**
(DOCX)

Text S2 **Easy tailoring of shape, size and materials.**
(DOCX)

Text S3 **Very large tunability.**
(DOCX)
