# Supplementary figures and images for: Water-Dispersible Three-Dimensional LC-Nanoresonators
Source: PLoS One. 2014 Aug 25;9(8):e105474. doi: 10.1371/journal.pone.0105474 (PMC4143276; doi:10.1371/journal.pone.0105474)

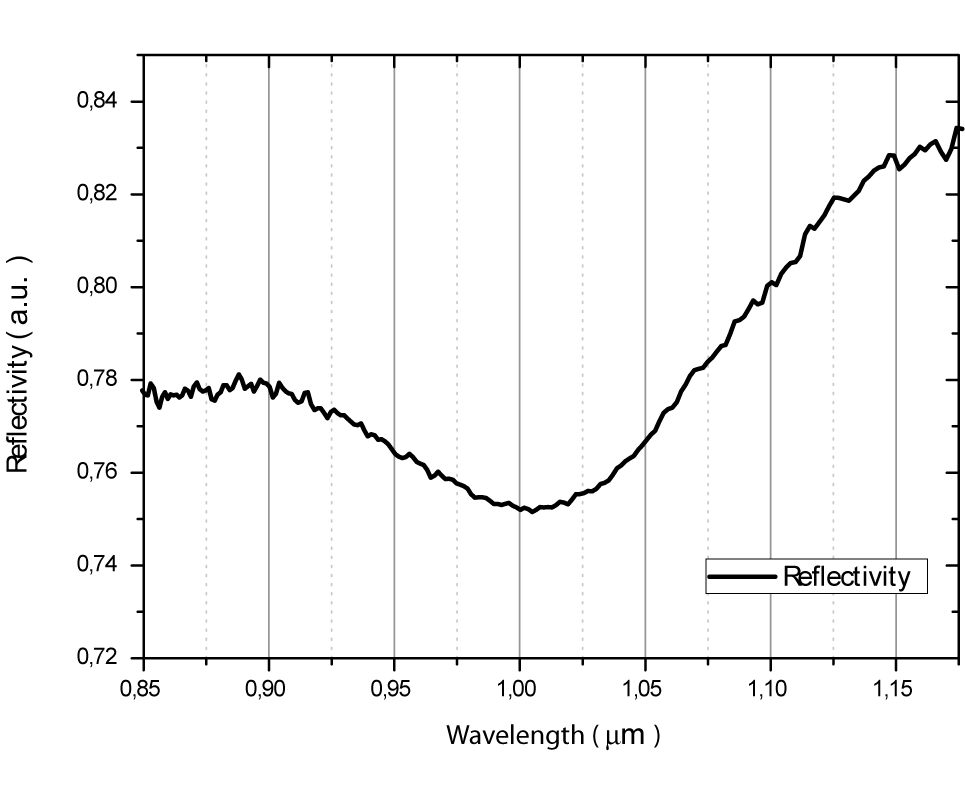

Supplement: Figure S1 — Characterization of pulled off nanoresonators. Reflectivity spectrum of pulled off nanoresonators. (TIF) [file pone.0105474.s001.tif]

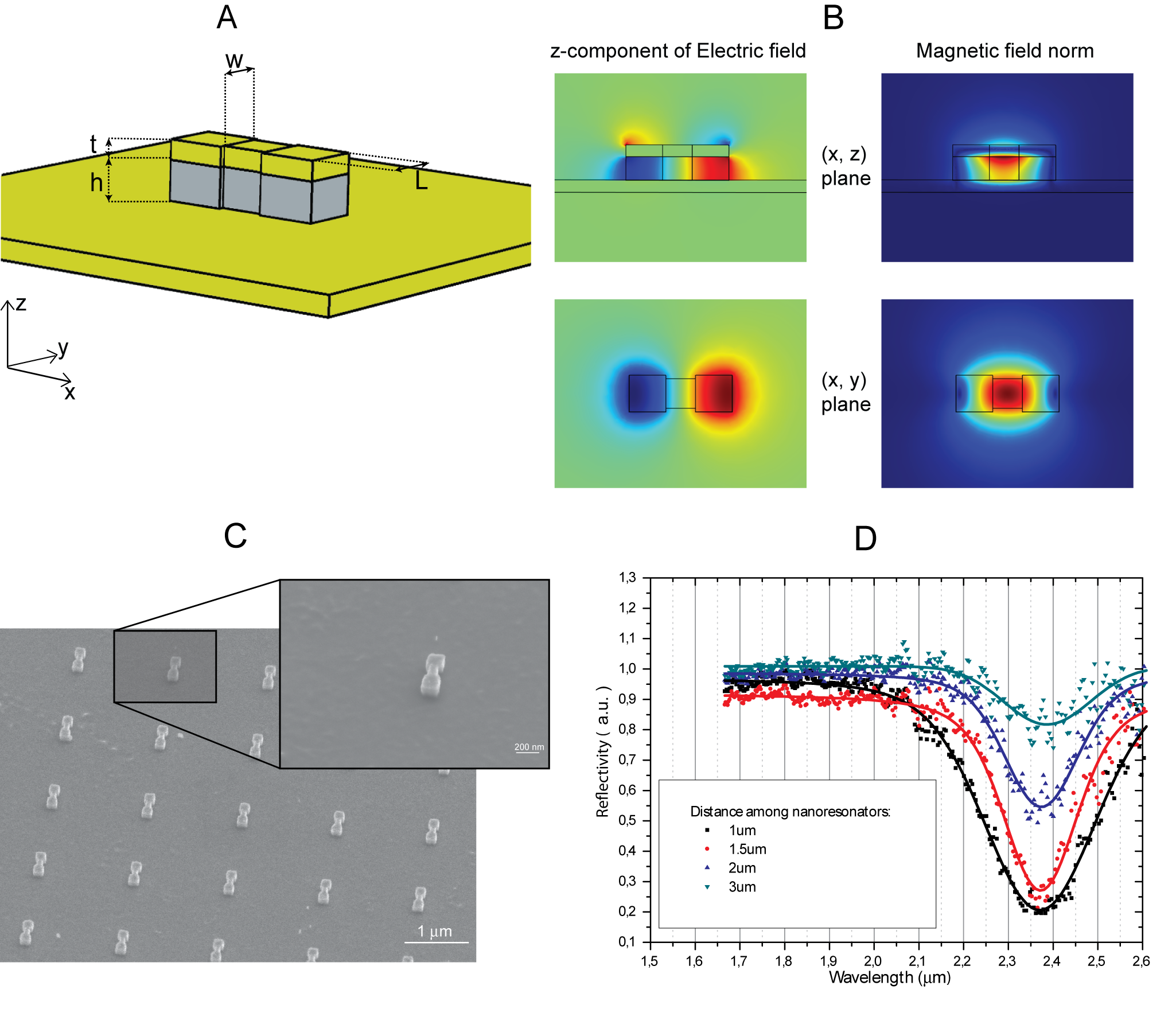

Supplement: Figure S2 — Bone shape nanoresonators. (A) Design of nanoresonators (B) Simulations of electric (z-component) and magnetic field (norm). The LC- behaviour is clearly visible (C) SEM image of nanoresonators disposed in array (D) Reflectivity of nanoresonators placed at different distances. (TIF) [file pone.0105474.s002.tif]

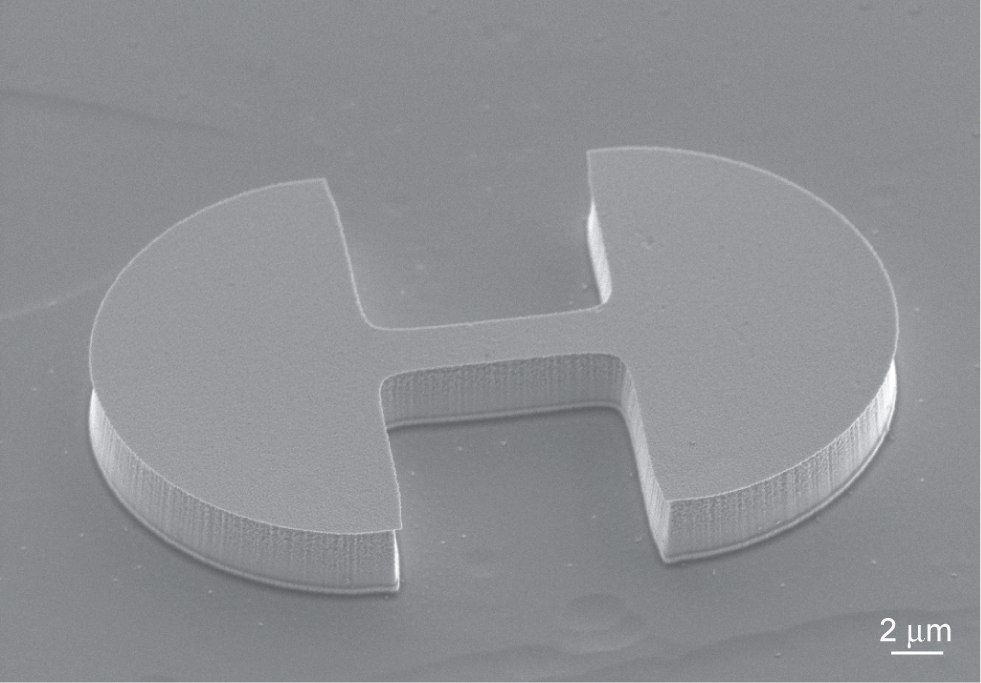

Supplement: Figure S3 — Thz LC nanoresonators. SEM image of LC nanoresonator for THz range. (TIF) [file pone.0105474.s003.tif]
