# Supplementary material for: Water-Dispersible Three-Dimensional LC-Nanoresonators
Source: PLoS One. 2014 Aug 25;9(8):e105474. doi: 10.1371/journal.pone.0105474 (PMC4143276; doi:10.1371/journal.pone.0105474)
Supplement: Table S1 — Figure 2C data. (PDF) [file pone.0105474.s004.pdf]

	A(X)	B(Y)	C(Y)	D(Y)
Long Name	Wavelength	Transmission		
Units	←m	(a.u.)		
Comments		nopol	M	0
1	2,00053	1,03281	1,03014	1,01983
2	1,99745	1,03286	1,02967	1,01993
3	1,99438	1,03288	1,02949	1,01989
4	1,99131	1,03258	1,02984	1,02028
5	1,98826	1,03239	1,02967	1,02041
6	1,98521	1,03258	1,02938	1,01989
7	1,98218	1,03291	1,0295	1,01978
8	1,97915	1,03292	1,02978	1,01995
9	1,97614	1,0327	1,02965	1,02013
10	1,97313	1,03259	1,02924	1,02027
11	1,97013	1,03268	1,02979	1,02021
12	1,96714	1,03282	1,03003	1,0204
13	1,96416	1,0328	1,02973	1,02038
14	1,96119	1,0327	1,02958	1,02049
15	1,95823	1,03272	1,02979	1,02042
16	1,95527	1,03275	1,03057	1,02052
17	1,95233	1,03279	1,03059	1,02043
18	1,94939	1,03271	1,03038	1,02016
19	1,94646	1,03272	1,03048	1,02004
20	1,94355	1,03281	1,0301	1,01969
21	1,94064	1,03292	1,02982	1,01959
22	1,93774	1,03289	1,03033	1,01988
23	1,93484	1,03279	1,03044	1,02013
24	1,93196	1,03285	1,03035	1,02041
25	1,92909	1,03297	1,02996	1,02038
26	1,92622	1,0329	1,02971	1,02028
27	1,92336	1,03285	1,02951	1,02019
28	1,92051	1,03302	1,02901	1,02024
29	1,91767	1,03288	1,02912	1,02002
30	1,91484	1,03297	1,02949	1,02006
31	1,91201	1,03293	1,02977	1,02053
32	1,9092	1,03294	1,03046	1,021
33	1,90639	1,03292	1,03068	1,0213
34	1,90359	1,03306	1,03	1,02103
35	1,9008	1,03356	1,02981	1,0208
36	1,89802	1,03327	1,02976	1,02013
37	1,89524	1,03269	1,02989	1,01985
38	1,89247	1,03268	1,02987	1,02043
39	1,88972	1,03273	1,02976	1,02069
40	1,88697	1,03259	1,03013	1,02083
41	1,88422	1,03276	1,03004	1,0206
42	1,88149	1,03269	1,02971	1,0199
43	1,87876	1,03267	1,02964	1,01955
44	1,87604	1,03298	1,02997	1,02061
45	1,87333	1,03325	1,0298	1,0221
46	1,87063	1,03298	1,02932	1,02168
47	1,86793	1,03268	1,02921	1,02042
48	1,86525	1,03242	1,02932	1,01984
49	1,86257	1,03256	1,02978	1,02011
50	1,85989	1,03275	1,03013	1,02055
51	1,85723	1,0326	1,02937	1,02024
52	1,85457	1,03286	1,02894	1,02067
53	1,85192	1,0328	1,0286	1,02025
54	1,84928	1,03288	1,02849	1,02019

	A(X)	B(Y)	C(Y)	D(Y)
Long Name	Wavelength	Transmission		
Units	←m	(a.u.)		
Comments		nopol	M	0
55	1,84665	1,03277	1,02899	1,02017
56	1,84402	1,03235	1,02954	1,02004
57	1,8414	1,03253	1,0297	1,02065
58	1,83879	1,03264	1,029	1,02063
59	1,83618	1,03237	1,02804	1,02024
60	1,83359	1,03202	1,02864	1,02057
61	1,831	1,03208	1,02922	1,02086
62	1,82841	1,03202	1,02895	1,02036
63	1,82584	1,03172	1,02837	1,01983
64	1,82327	1,03186	1,02806	1,01968
65	1,82071	1,03213	1,02837	1,02023
66	1,81816	1,03206	1,02803	1,01968
67	1,81561	1,03198	1,02748	1,01928
68	1,81307	1,03162	1,02758	1,01905
69	1,81054	1,03161	1,02831	1,01916
70	1,80801	1,03161	1,02812	1,01951
71	1,8055	1,03144	1,02755	1,01924
72	1,80298	1,0315	1,0276	1,01901
73	1,80048	1,03148	1,02766	1,0191
74	1,79798	1,03136	1,02757	1,01954
75	1,79549	1,03122	1,02732	1,01979
76	1,79301	1,03128	1,02724	1,01917
77	1,79053	1,03136	1,02722	1,01883
78	1,78806	1,0312	1,02736	1,01909
79	1,7856	1,03096	1,02782	1,01915
80	1,78314	1,03093	1,02777	1,01905
81	1,78069	1,031	1,02729	1,01902
82	1,77825	1,03083	1,02649	1,01883
83	1,77582	1,03087	1,02629	1,01865
84	1,77339	1,0309	1,02676	1,01869
85	1,77096	1,03085	1,02675	1,01841
86	1,76855	1,03088	1,02662	1,01822
87	1,76614	1,03078	1,02655	1,0188
88	1,76373	1,03076	1,02677	1,01913
89	1,76134	1,03058	1,02683	1,01882
90	1,75895	1,03068	1,02629	1,01883
91	1,75657	1,03077	1,02651	1,01919
92	1,75419	1,03067	1,02655	1,0187
93	1,75182	1,03052	1,02583	1,01808
94	1,74945	1,03048	1,0254	1,01794
95	1,7471	1,03062	1,02541	1,0181
96	1,74474	1,03046	1,02554	1,01815
97	1,7424	1,03039	1,02595	1,01795
98	1,74006	1,03047	1,02573	1,01774
99	1,73773	1,03041	1,02495	1,0178
100	1,7354	1,03003	1,02499	1,01806
101	1,73308	1,02988	1,02528	1,01807
102	1,73077	1,02988	1,025	1,01779
103	1,72846	1,02964	1,02502	1,01773
104	1,72616	1,02973	1,02564	1,018
105	1,72386	1,02987	1,02562	1,01793
106	1,72157	1,02991	1,02494	1,01764
107	1,71929	1,02983	1,02468	1,01783
108	1,71701	1,02967	1,02466	1,0179
109	1,71474	1,02971	1,02419	1,01788
110	1,71248	1,02984	1,02408	1,01779
111	1,71022	1,02985	1,02429	1,01776

	A(X)	B(Y)	C(Y)	D(Y)
Long Name	Wavelength	Transmission		
Units	←m	(a.u.)		
Comments		nopol	M	0
112	1,70796	1,02966	1,02452	1,01774
113	1,70572	1,02941	1,02447	1,01786
114	1,70348	1,02939	1,02424	1,01792
115	1,70124	1,02939	1,02428	1,01759
116	1,69901	1,02908	1,0242	1,01732
117	1,69679	1,02894	1,02387	1,01692
118	1,69457	1,02916	1,02361	1,01676
119	1,69236	1,02932	1,02398	1,01683
120	1,69015	1,02923	1,02424	1,01685
121	1,68795	1,02918	1,02404	1,01693
122	1,68575	1,02924	1,02405	1,01679
123	1,68357	1,02915	1,02402	1,01692
124	1,68138	1,02896	1,02385	1,01685
125	1,6792	1,02885	1,02366	1,01691
126	1,67703	1,02864	1,02337	1,01691
127	1,67486	1,02863	1,02319	1,01656
128	1,6727	1,02857	1,02306	1,01628
129	1,67055	1,02858	1,02344	1,01648
130	1,6684	1,02843	1,0233	1,01654
131	1,66625	1,02813	1,02302	1,01625
132	1,66411	1,02804	1,02256	1,01595
133	1,66198	1,028	1,02229	1,01614
134	1,65985	1,02785	1,02248	1,01663
135	1,65773	1,02782	1,02238	1,01637
136	1,65561	1,02787	1,02225	1,01616
137	1,6535	1,02761	1,02228	1,01618
138	1,6514	1,02763	1,02195	1,01606
139	1,64929	1,02776	1,02164	1,0159
140	1,6472	1,02779	1,02154	1,01563
141	1,64511	1,02771	1,02128	1,01578
142	1,64302	1,02751	1,02155	1,01622
143	1,64094	1,02719	1,02224	1,016
144	1,63887	1,027	1,02209	1,01558
145	1,6368	1,02704	1,02124	1,01539
146	1,63474	1,02697	1,02085	1,01514
147	1,63268	1,02684	1,0212	1,01516
148	1,63062	1,02679	1,02099	1,01587
149	1,62857	1,02677	1,02064	1,01602
150	1,62653	1,02671	1,02071	1,01572
151	1,62449	1,02663	1,02036	1,01565
152	1,62246	1,02644	1,01938	1,01552
153	1,62043	1,02618	1,01932	1,01538
154	1,61841	1,02629	1,0196	1,01565
155	1,61639	1,02616	1,01911	1,01509
156	1,61438	1,02593	1,01917	1,01489
157	1,61237	1,02595	1,01968	1,01546
158	1,61037	1,026	1,01999	1,01493
159	1,60837	1,02577	1,01962	1,01457
160	1,60638	1,02561	1,01902	1,01457
161	1,60439	1,02565	1,0192	1,015
162	1,6024	1,02554	1,01902	1,01515
163	1,60043	1,02536	1,01844	1,01444
164	1,59845	1,02532	1,01858	1,01407
165	1,59648	1,02517	1,0185	1,01396
166	1,59452	1,02486	1,01762	1,01421
167	1,59256	1,02493	1,01745	1,0145
168	1,59061	1,02488	1,01814	1,01446
169	1,58866	1,02459	1,01803	1,01401

	A(X)	B(Y)	C(Y)	D(Y)
Long Name	Wavelength	Transmission		
Units	←m	(a.u.)		
Comments		nopol	M	0
170	1,58671	1,02449	1,01768	1,01392
171	1,58477	1,02462	1,01747	1,01389
172	1,58284	1,0245	1,01744	1,01422
173	1,58091	1,02417	1,0176	1,01439
174	1,57898	1,02407	1,01713	1,01437
175	1,57706	1,0241	1,01664	1,014
176	1,57515	1,02417	1,01632	1,01371
177	1,57323	1,02421	1,01634	1,01408
178	1,57133	1,02391	1,01649	1,01429
179	1,56943	1,02351	1,01681	1,01389
180	1,56753	1,02332	1,01625	1,01369
181	1,56563	1,02321	1,01544	1,01372
182	1,56375	1,02318	1,01553	1,01379
183	1,56186	1,02317	1,01506	1,01368
184	1,55998	1,02289	1,01475	1,01381
185	1,55811	1,02257	1,015	1,01379
186	1,55624	1,0225	1,01486	1,01367
187	1,55437	1,02245	1,01462	1,01367
188	1,55251	1,0222	1,01397	1,01372
189	1,55065	1,02198	1,0139	1,01344
190	1,5488	1,02178	1,01365	1,01293
191	1,54695	1,02167	1,01275	1,01253
192	1,54511	1,02149	1,01245	1,01288
193	1,54327	1,0213	1,01234	1,01336
194	1,54143	1,02151	1,01228	1,01308
195	1,5396	1,02156	1,01176	1,01264
196	1,53778	1,02148	1,01142	1,01224
197	1,53595	1,02122	1,0112	1,01248
198	1,53414	1,02109	1,01131	1,01254
199	1,53232	1,02094	1,01158	1,0121
200	1,53051	1,02054	1,01126	1,01189
201	1,52871	1,02012	1,01068	1,0121
202	1,52691	1,02001	1,01074	1,01197
203	1,52511	1,01983	1,01051	1,01185
204	1,52332	1,0197	1,00967	1,01179
205	1,52153	1,01999	1,009	1,01155
206	1,51975	1,01991	1,00896	1,01188
207	1,51797	1,01962	1,00888	1,01215
208	1,51619	1,01934	1,00827	1,01169
209	1,51442	1,01888	1,0081	1,01155
210	1,51265	1,01861	1,00825	1,01168
211	1,51089	1,01837	1,00794	1,01119
212	1,50913	1,01826	1,00745	1,01088
213	1,50738	1,01819	1,00744	1,01078
214	1,50563	1,01786	1,00721	1,01106
215	1,50388	1,01772	1,00639	1,01127
216	1,50214	1,01757	1,006	1,0111
217	1,5004	1,0173	1,00623	1,01054
218	1,49867	1,01705	1,00543	1,00993
219	1,49693	1,01667	1,00466	1,01044
220	1,49521	1,01633	1,00437	1,0105
221	1,49349	1,01639	1,00397	1,01049
222	1,49177	1,01615	1,00423	1,01127
223	1,49005	1,01618	1,00404	1,01112
224	1,48834	1,01639	1,0034	1,01059
225	1,48663	1,016	1,0034	1,01062
226	1,48493	1,01543	1,00287	1,00974
227	1,48323	1,01503	1,0022	1,00914

	A(X)	B(Y)	C(Y)	D(Y)
Long Name	Wavelength	Transmission		
Units	←m	(a.u.)		
Comments		nopol	M	0
228	1,48154	1,01506	1,00184	1,00883
229	1,47985	1,01489	1,00083	1,0089
230	1,47816	1,01461	1,00067	1,0095
231	1,47648	1,01449	1,00058	1,00982
232	1,4748	1,01405	1,00039	1,00949
233	1,47312	1,01358	0,99977	1,00973
234	1,47145	1,01354	0,99951	1,00975
235	1,46978	1,01329	0,99922	1,00884
236	1,46811	1,01302	0,99903	1,00846
237	1,46645	1,01287	0,9989	1,00896
238	1,4648	1,01229	0,99801	1,0094
239	1,46314	1,01175	0,99688	1,00965
240	1,46149	1,01163	0,99595	1,00953
241	1,45985	1,01148	0,99607	1,00932
242	1,45821	1,01135	0,99593	1,00867
243	1,45657	1,01119	0,99517	1,00796
244	1,45493	1,01035	0,99462	1,00777
245	1,4533	1,00967	0,99403	1,00742
246	1,45167	1,0097	0,99329	1,00773
247	1,45005	1,00974	0,99324	1,00832
248	1,44843	1,00963	0,9927	1,00845
249	1,44681	1,00936	0,99187	1,00835
250	1,4452	1,00904	0,99134	1,00763
251	1,44359	1,00892	0,99145	1,00697
252	1,44199	1,00861	0,99099	1,00718
253	1,44038	1,00822	0,98988	1,00704
254	1,43878	1,00776	0,99039	1,00712
255	1,43719	1,00745	0,99021	1,00744
256	1,4356	1,00732	0,98922	1,00723
257	1,43401	1,00696	0,98899	1,00675
258	1,43243	1,00648	0,98862	1,00619
259	1,43084	1,00612	0,98715	1,00607
260	1,42927	1,00596	0,98683	1,00631
261	1,42769	1,00566	0,98674	1,00634
262	1,42612	1,00527	0,98588	1,00629
263	1,42456	1,00513	0,98492	1,00594
264	1,42299	1,0049	0,98394	1,00517
265	1,42143	1,00482	0,98337	1,00539
266	1,41987	1,00457	0,98321	1,00545
267	1,41832	1,00384	0,98282	1,00519
268	1,41677	1,00309	0,9828	1,00487
269	1,41522	1,00323	0,98251	1,00498
270	1,41368	1,00266	0,98138	1,00549
271	1,41214	1,00194	0,98066	1,00518
272	1,4106	1,00218	0,98005	1,00471
273	1,40907	1,00214	0,97913	1,00473
274	1,40754	1,00154	0,9795	1,0047
275	1,40601	1,00126	0,97897	1,00498
276	1,40449	1,00094	0,97762	1,00443
277	1,40297	1,00014	0,97769	1,00347
278	1,40145	1,00003	0,97749	1,00405
279	1,39994	0,99996	0,97622	1,00384
280	1,39843	0,99974	0,97584	1,00311
281	1,39692	0,99921	0,97527	1,00368
282	1,39542	0,99855	0,97403	1,00393
283	1,39392	0,99815	0,97393	1,00333
284	1,39242	0,9977	0,97386	1,00324
285	1,39093	0,99688	0,97314	1,00323

	A(X)	B(Y)	C(Y)	D(Y)
Long Name	Wavelength	Transmission		
Units	←m	(a.u.)		
Comments		nopol	M	0
286	1,38944	0,99645	0,97197	1,00284
287	1,38795	0,99654	0,97214	1,0024
288	1,38647	0,99647	0,97128	1,00195
289	1,38498	0,99594	0,96965	1,00269
290	1,38351	0,99535	0,96896	1,00338
291	1,38203	0,99476	0,9691	1,00335
292	1,38056	0,99424	0,96811	1,0028
293	1,37909	0,99369	0,96672	1,00184
294	1,37763	0,99308	0,96579	1,00143
295	1,37616	0,9926	0,96508	1,00164
296	1,3747	0,99253	0,96375	1,0016
297	1,37325	0,99208	0,96299	1,00121
298	1,37179	0,99169	0,96218	1,00115
299	1,37034	0,9913	0,9622	1,00173
300	1,3689	0,99073	0,96233	1,0015
301	1,36745	0,9902	0,96121	1,00067
302	1,36601	0,98984	0,96059	1,00038
303	1,36457	0,9897	0,96001	1,00064
304	1,36314	0,98982	0,95913	1,00071
305	1,36171	0,98956	0,95845	1,0012
306	1,36028	0,98826	0,95722	1,00087
307	1,35885	0,98721	0,95536	0,99968
308	1,35743	0,98673	0,95425	0,9987
309	1,35601	0,98612	0,95438	0,9986
310	1,35459	0,98571	0,95322	0,9983
311	1,35318	0,98532	0,95094	0,9986
312	1,35177	0,98436	0,95044	0,99856
313	1,35036	0,98362	0,9503	0,99738
314	1,34895	0,98314	0,9489	0,99765
315	1,34755	0,98279	0,94776	0,99862
316	1,34615	0,98199	0,94715	0,99828
317	1,34476	0,98133	0,94598	0,99773
318	1,34336	0,98066	0,94455	0,99756
319	1,34197	0,97991	0,94369	0,9973
320	1,34058	0,97937	0,94283	0,99713
321	1,3392	0,97896	0,94154	0,9965
322	1,33782	0,97827	0,94021	0,9954
323	1,33644	0,97763	0,93949	0,99505
324	1,33506	0,97707	0,93873	0,99554
325	1,33369	0,9764	0,93852	0,99607
326	1,33232	0,97564	0,93656	0,99603
327	1,33095	0,9749	0,93413	0,99543
328	1,32958	0,97395	0,93284	0,99419
329	1,32822	0,97322	0,93195	0,99392
330	1,32686	0,97248	0,93135	0,99409
331	1,32551	0,97167	0,93073	0,99377
332	1,32415	0,97101	0,92985	0,99301
333	1,3228	0,97028	0,92862	0,99292
334	1,32145	0,96934	0,92726	0,99304
335	1,32011	0,96859	0,92483	0,99332
336	1,31876	0,96809	0,92347	0,99287
337	1,31742	0,96733	0,92331	0,99247
338	1,31609	0,96673	0,92254	0,99251
339	1,31475	0,9664	0,92058	0,9925
340	1,31342	0,96586	0,91909	0,99237
341	1,31209	0,96454	0,91773	0,99228
342	1,31076	0,96354	0,9166	0,99143
343	1,30944	0,96306	0,91571	0,99008

	A(X)	B(Y)	C(Y)	D(Y)
Long Name	Wavelength	Transmission		
Units	←m	(a.u.)		
Comments		nopol	M	0
344	1,30812	0,96228	0,91372	0,99006
345	1,3068	0,96087	0,91028	0,99116
346	1,30548	0,9601	0,90836	0,99155
347	1,30417	0,9599	0,90828	0,98989
348	1,30286	0,95918	0,90813	0,98833
349	1,30155	0,95786	0,90669	0,98865
350	1,30025	0,95674	0,90458	0,98909
351	1,29894	0,95594	0,9027	0,98812
352	1,29764	0,95519	0,90095	0,98727
353	1,29635	0,95383	0,89936	0,98692
354	1,29505	0,95246	0,89792	0,98722
355	1,29376	0,9516	0,89712	0,98745
356	1,29247	0,95077	0,89538	0,9865
357	1,29118	0,94982	0,89324	0,98704
358	1,2899	0,94817	0,89141	0,9877
359	1,28861	0,94676	0,88967	0,98622
360	1,28733	0,94635	0,88816	0,98477
361	1,28606	0,94583	0,8864	0,98441
362	1,28478	0,94476	0,88348	0,98411
363	1,28351	0,94344	0,88157	0,98307
364	1,28224	0,94238	0,88026	0,98262
365	1,28097	0,94126	0,87854	0,98282
366	1,27971	0,94014	0,87641	0,98246
367	1,27845	0,9386	0,87424	0,98161
368	1,27719	0,93753	0,87251	0,9807
369	1,27593	0,93699	0,87129	0,97976
370	1,27468	0,93586	0,86933	0,97964
371	1,27342	0,93456	0,86741	0,98018
372	1,27217	0,93292	0,86558	0,98031
373	1,27093	0,93166	0,86305	0,9799
374	1,26968	0,93066	0,86083	0,97922
375	1,26844	0,92925	0,85928	0,97873
376	1,2672	0,92813	0,85905	0,9786
377	1,26596	0,92717	0,85686	0,97805
378	1,26473	0,92611	0,85411	0,97733
379	1,26349	0,92479	0,85286	0,97701
380	1,26226	0,92368	0,85074	0,97691
381	1,26104	0,92265	0,8478	0,97664
382	1,25981	0,92126	0,84565	0,97591
383	1,25859	0,91989	0,84432	0,97582
384	1,25737	0,91864	0,84308	0,97572
385	1,25615	0,91785	0,84091	0,97422
386	1,25493	0,91698	0,8388	0,97364
387	1,25372	0,91568	0,83688	0,97397
388	1,25251	0,91402	0,83529	0,9737
389	1,2513	0,9127	0,83374	0,97284
390	1,25009	0,91168	0,83253	0,97215
391	1,24889	0,91056	0,83037	0,97267
392	1,24769	0,90948	0,82779	0,97195
393	1,24649	0,90839	0,82536	0,97085
394	1,24529	0,90736	0,82366	0,97024
395	1,24409	0,90586	0,82242	0,96955
396	1,2429	0,90487	0,82104	0,96889
397	1,24171	0,90388	0,81985	0,96769
398	1,24052	0,90261	0,81818	0,96722
399	1,23934	0,90179	0,81622	0,96735
400	1,23815	0,90052	0,81463	0,96775
401	1,23697	0,89934	0,81206	0,96769

	A(X)	B(Y)	C(Y)	D(Y)
Long Name	Wavelength	Transmission		
Units	←m	(a.u.)		
Comments		nopol	M	0
402	1,23579	0,89837	0,80981	0,96647
403	1,23461	0,89734	0,80943	0,96535
404	1,23344	0,89644	0,80844	0,96443
405	1,23227	0,89532	0,80701	0,96457
406	1,2311	0,89427	0,80656	0,96602
407	1,22993	0,89316	0,80559	0,96565
408	1,22876	0,89179	0,8043	0,96442
409	1,2276	0,89106	0,8035	0,96355
410	1,22644	0,89097	0,80187	0,96268
411	1,22528	0,88963	0,79967	0,96178
412	1,22412	0,88805	0,79818	0,96104
413	1,22297	0,88749	0,79781	0,95998
414	1,22181	0,88678	0,79764	0,96006
415	1,22066	0,88579	0,7966	0,9603
416	1,21952	0,88453	0,7959	0,96114
417	1,21837	0,88396	0,79476	0,96071
418	1,21723	0,8837	0,7926	0,95909
419	1,21608	0,88331	0,79238	0,95865
420	1,21494	0,88267	0,79316	0,95871
421	1,21381	0,8813	0,7925	0,95863
422	1,21267	0,88047	0,79088	0,95844
423	1,21154	0,88048	0,78911	0,95808
424	1,21041	0,88012	0,78888	0,95724
425	1,20928	0,87957	0,78791	0,95662
426	1,20815	0,87896	0,78767	0,95627
427	1,20703	0,8782	0,78761	0,95479
428	1,2059	0,87773	0,7869	0,95476
429	1,20478	0,87715	0,78619	0,95551
430	1,20366	0,87688	0,78779	0,95501
431	1,20255	0,87685	0,78703	0,95443
432	1,20143	0,87621	0,78612	0,95383
433	1,20032	0,87575	0,78505	0,95354
434	1,19921	0,87538	0,78507	0,9532
435	1,1981	0,8746	0,78483	0,95236
436	1,19699	0,87432	0,78503	0,95158
437	1,19589	0,87454	0,78512	0,95224
438	1,19479	0,87463	0,78372	0,95286
439	1,19369	0,87392	0,78333	0,95269
440	1,19259	0,87315	0,7839	0,95259
441	1,19149	0,87228	0,78336	0,95313
442	1,1904	0,8723	0,78379	0,95203
443	1,18931	0,87236	0,78361	0,94973
444	1,18822	0,87154	0,78324	0,9502
445	1,18713	0,87084	0,7841	0,95129
446	1,18604	0,87087	0,78381	0,9506
447	1,18496	0,87078	0,78231	0,94928
448	1,18388	0,87052	0,78213	0,94874
449	1,1828	0,87023	0,78206	0,95038
450	1,18172	0,86963	0,7826	0,95017
451	1,18064	0,86935	0,7836	0,94888
452	1,17957	0,86948	0,78194	0,9473
453	1,1785	0,86933	0,78144	0,94638
454	1,17743	0,86842	0,78214	0,94641
455	1,17636	0,86833	0,78149	0,94542
456	1,17529	0,86867	0,78082	0,94498
457	1,17423	0,86788	0,78164	0,94569
458	1,17316	0,86688	0,78178	0,9461
459	1,1721	0,86615	0,78269	0,94606

	A(X)	B(Y)	C(Y)	D(Y)
Long Name	Wavelength	Transmission		
Units	←m	(a.u.)		
Comments		nopol	M	0
460	1,17104	0,86564	0,78242	0,94482
461	1,16999	0,86513	0,78279	0,94412
462	1,16893	0,86553	0,78307	0,9449
463	1,16788	0,86578	0,78146	0,94534
464	1,16683	0,86575	0,78124	0,94325
465	1,16578	0,86523	0,78158	0,94115
466	1,16473	0,86418	0,78057	0,94114
467	1,16369	0,86326	0,78042	0,94153
468	1,16264	0,86287	0,78149	0,94023
469	1,1616	0,86266	0,78067	0,93936
470	1,16056	0,86226	0,77995	0,93971
471	1,15952	0,86191	0,77994	0,94091
472	1,15849	0,86219	0,77964	0,94147
473	1,15745	0,86163	0,77864	0,94046
474	1,15642	0,86122	0,77815	0,93957
475	1,15539	0,86066	0,77778	0,93996
476	1,15436	0,86008	0,77727	0,94011
477	1,15333	0,85959	0,77674	0,93951
478	1,15231	0,85922	0,77656	0,93935
479	1,15128	0,85862	0,77661	0,93858
480	1,15026	0,85774	0,77607	0,93734
481	1,14924	0,85673	0,77492	0,93731
482	1,14822	0,85591	0,77531	0,93677
483	1,14721	0,85561	0,77483	0,93554
484	1,14619	0,85513	0,77372	0,93554
485	1,14518	0,85383	0,77198	0,93614
486	1,14417	0,85339	0,77097	0,93483
487	1,14316	0,85294	0,77079	0,93354
488	1,14215	0,85159	0,7693	0,93268
489	1,14115	0,85076	0,76822	0,93244
490	1,14015	0,85094	0,76916	0,93177
491	1,13914	0,85032	0,76868	0,93146
492	1,13814	0,84917	0,76645	0,93247
493	1,13715	0,84848	0,76594	0,93215
494	1,13615	0,84746	0,76542	0,93014
495	1,13515	0,84659	0,76489	0,92931
496	1,13416	0,84616	0,76418	0,92902
497	1,13317	0,84579	0,76331	0,92866
498	1,13218	0,84473	0,7619	0,92837
499	1,13119	0,84313	0,75996	0,92688
500	1,13021	0,84241	0,75987	0,92742
501	1,12922	0,84121	0,75967	0,92702
502	1,12824	0,84014	0,75899	0,92488
503	1,12726	0,83962	0,75795	0,92501
504	1,12628	0,83858	0,75598	0,92507
505	1,1253	0,83724	0,75381	0,92359
506	1,12432	0,83593	0,75424	0,92311
507	1,12335	0,83491	0,75226	0,92357
508	1,12238	0,83471	0,7508	0,92095
509	1,12141	0,83373	0,75067	0,91875
510	1,12044	0,83298	0,75089	0,91952
511	1,11947	0,83199	0,74917	0,91942
512	1,1185	0,82974	0,74746	0,91883
513	1,11754	0,82812	0,74649	0,91944
514	1,11658	0,82793	0,74483	0,92062
515	1,11562	0,82737	0,74282	0,91779
516	1,11466	0,82622	0,74207	0,91567
517	1,1137	0,82479	0,74075	0,91569

	A(X)	B(Y)	C(Y)	D(Y)
Long Name	Wavelength	Transmission		
Units	←m	(a.u.)		
Comments		nopol	M	0
518	1,11274	0,82304	0,73947	0,91564
519	1,11179	0,82198	0,73837	0,91575
520	1,11084	0,82114	0,73761	0,91628
521	1,10988	0,81977	0,73535	0,91531
522	1,10894	0,81843	0,73363	0,91376
523	1,10799	0,81713	0,73217	0,91235
524	1,10704	0,81547	0,73168	0,91131
525	1,1061	0,81407	0,72994	0,90968
526	1,10515	0,81327	0,72811	0,91035
527	1,10421	0,81234	0,72588	0,91008
528	1,10327	0,81091	0,72357	0,90817
529	1,10233	0,80873	0,72329	0,90687
530	1,1014	0,80721	0,72407	0,90605
531	1,10046	0,80667	0,72251	0,90565
532	1,09953	0,80618	0,7194	0,90521
533	1,0986	0,80591	0,71703	0,90541
534	1,09767	0,80478	0,7163	0,9048
535	1,09674	0,80279	0,7141	0,90397
536	1,09581	0,80105	0,71321	0,90354
537	1,09489	0,79995	0,71266	0,90301
538	1,09396	0,7989	0,71117	0,90284
539	1,09304	0,79806	0,71016	0,90124
540	1,09212	0,79669	0,70767	0,89805
541	1,0912	0,79563	0,70553	0,89774
542	1,09028	0,7958	0,70561	0,89813
543	1,08937	0,79443	0,70595	0,89845
544	1,08845	0,79311	0,70585	0,89823
545	1,08754	0,79209	0,70405	0,89729
546	1,08663	0,79145	0,70052	0,89608
547	1,08572	0,7904	0,69854	0,89413
548	1,08481	0,78984	0,6984	0,89565
549	1,0839	0,78869	0,69802	0,89644
550	1,083	0,7872	0,69777	0,89479
551	1,08209	0,78554	0,69802	0,89296
552	1,08119	0,78556	0,69823	0,89255
553	1,08029	0,78556	0,69552	0,89216
554	1,07939	0,78418	0,6933	0,89125
555	1,07849	0,78336	0,69362	0,89172
556	1,07759	0,78293	0,69437	0,89089
557	1,0767	0,78207	0,6929	0,88864
558	1,07581	0,78171	0,6921	0,88882
559	1,07491	0,78173	0,6932	0,88907
560	1,07402	0,78144	0,69321	0,88932
561	1,07313	0,78127	0,6926	0,88929
562	1,07225	0,78135	0,69335	0,88768
563	1,07136	0,78101	0,69332	0,88714
564	1,07048	0,78031	0,69202	0,88735
565	1,06959	0,77942	0,69135	0,88652
566	1,06871	0,77898	0,69202	0,88748
567	1,06783	0,77899	0,6924	0,8871
568	1,06695	0,77916	0,69281	0,88658
569	1,06607	0,77916	0,69332	0,88642
570	1,0652	0,77981	0,69413	0,88575
571	1,06432	0,78015	0,69445	0,88534
572	1,06345	0,77961	0,69272	0,88498
573	1,06258	0,77951	0,69227	0,88487
574	1,06171	0,77917	0,69562	0,88553
575	1,06084	0,77929	0,69592	0,88559

	A(X)	B(Y)	C(Y)	D(Y)
Long Name	Wavelength	Transmission		
Units	←m	(a.u.)		
Comments		nopol	M	0
576	1,05997	0,77958	0,69419	0,88442
577	1,05911	0,77999	0,69436	0,88319
578	1,05824	0,78016	0,69738	0,88325
579	1,05738	0,78004	0,69779	0,88485
580	1,05652	0,78045	0,69676	0,88547
581	1,05566	0,78097	0,6982	0,8873
582	1,0548	0,78188	0,69942	0,88618
583	1,05394	0,78215	0,69894	0,88457
584	1,05308	0,78193	0,69856	0,88527
585	1,05223	0,78156	0,70129	0,88422
586	1,05138	0,78204	0,70231	0,8834
587	1,05052	0,78348	0,7027	0,88522
588	1,04967	0,78514	0,70466	0,88524
589	1,04882	0,78462	0,70609	0,8833
590	1,04798	0,78426	0,70694	0,88266
591	1,04713	0,78521	0,70781	0,88131
592	1,04628	0,78562	0,70893	0,88172
593	1,04544	0,78617	0,70931	0,88242
594	1,0446	0,78694	0,71003	0,88322
595	1,04376	0,78764	0,71056	0,88301
596	1,04292	0,788	0,71259	0,88418
597	1,04208	0,78903	0,71443	0,88506
598	1,04124	0,79027	0,71613	0,88335
599	1,04041	0,79086	0,71743	0,88139
600	1,03957	0,79208	0,7187	0,8808
601	1,03874	0,79255	0,71934	0,88029
602	1,03791	0,79223	0,7203	0,88268
603	1,03708	0,79254	0,7227	0,88594
604	1,03625	0,79457	0,72324	0,88591
605	1,03542	0,79466	0,7241	0,88469
606	1,03459	0,79426	0,72498	0,88418
607	1,03377	0,79478	0,72567	0,88439
608	1,03294	0,79519	0,72862	0,88487
609	1,03212	0,79611	0,73085	0,88628
610	1,0313	0,79821	0,73051	0,88427
611	1,03048	0,80006	0,73184	0,88271
612	1,02966	0,8011	0,73355	0,88322
613	1,02885	0,80089	0,7329	0,88191
614	1,02803	0,80087	0,73547	0,8813
615	1,02721	0,80162	0,73687	0,88278
616	1,0264	0,80227	0,73698	0,88293
617	1,02559	0,80331	0,73835	0,88207
618	1,02478	0,80493	0,74064	0,88052
619	1,02397	0,80634	0,74426	0,88101
620	1,02316	0,80584	0,74747	0,88224
621	1,02235	0,80608	0,74766	0,88237
622	1,02155	0,8074	0,74672	0,88263
623	1,02074	0,80851	0,74685	0,88262
624	1,01994	0,80949	0,74534	0,88283
625	1,01914	0,8099	0,74575	0,88406
626	1,01834	0,81136	0,7494	0,88292
627	1,01754	0,81201	0,75	0,88178
628	1,01674	0,81252	0,74979	0,88151
629	1,01594	0,81327	0,74966	0,8835
630	1,01515	0,81352	0,75195	0,88202
631	1,01435	0,81505	0,75423	0,8798
632	1,01356	0,81654	0,75638	0,88327
633	1,01277	0,81643	0,75763	0,88444

	A(X)	B(Y)	C(Y)	D(Y)
Long Name	Wavelength	Transmission		
Units	←m	(a.u.)		
Comments		nopol	M	0
634	1,01198	0,81567	0,75921	0,88161
635	1,01119	0,81642	0,76292	0,88
636	1,0104	0,81852	0,76434	0,87706
637	1,00961	0,81885	0,76355	0,87768
638	1,00883	0,81868	0,76286	0,87785
639	1,00804	0,81953	0,76277	0,87616
640	1,00726	0,8206	0,76286	0,87699
641	1,00648	0,82259	0,76506	0,87705
642	1,0057	0,82428	0,76724	0,87675
643	1,00492	0,82311	0,7693	0,87497
644	1,00414	0,82234	0,77168	0,87511
645	1,00336	0,82352	0,77147	0,87629
646	1,00259	0,82481	0,76859	0,87628
647	1,00181	0,82513	0,76981	0,87422
648	1,00104	0,82625	0,77404	0,87628
649	1,00027	0,82605	0,77498	0,87693
650	0,9995	0,82618	0,77356	0,87591
651	0,99873	0,82721	0,77219	0,8767
652	0,99796	0,82836	0,77357	0,87593
653	0,99719	0,82811	0,77636	0,87524
654	0,99642	0,82688	0,77659	0,87477
655	0,99566	0,82682	0,77721	0,87065
656	0,99489	0,82882	0,77664	0,86853
657	0,99413	0,83046	0,77398	0,86806
658	0,99337	0,83139	0,77457	0,8693
659	0,99261	0,83199	0,7762	0,86975
660	0,99185	0,83135	0,77708	0,8667
661	0,99109	0,83059	0,77886	0,86596
662	0,99033	0,83098	0,77992	0,86581
663	0,98958	0,83198	0,78014	0,86513
664	0,98882	0,83201	0,7827	0,8649
665	0,98807	0,83099	0,78461	0,86661
666	0,98732	0,8299	0,78123	0,86999
667	0,98656	0,83158	0,77885	0,86785
668	0,98581	0,83415	0,78133	0,86477
669	0,98506	0,83447	0,78108	0,86515
670	0,98432	0,83463	0,78168	0,86345
671	0,98357	0,83495	0,78105	0,8635
672	0,98282	0,83674	0,78129	0,86242
673	0,98208	0,83753	0,78368	0,85982
674	0,98134	0,83751	0,78344	0,86039
675	0,98059	0,83678	0,78249	0,85667
676	0,97985	0,83773	0,78179	0,85328
677	0,97911	0,83874	0,7829	0,85573
678	0,97837	0,83906	0,78475	0,85496
679	0,97764	0,84046	0,78453	0,85327
680	0,9769	0,84131	0,7834	0,85282
681	0,97616	0,84144	0,78122	0,85109
682	0,97543	0,84096	0,77993	0,84969
683	0,9747	0,84153	0,78391	0,85045
684	0,97396	0,84364	0,78526	0,84974
685	0,97323	0,8447	0,78551	0,84754
686	0,9725	0,84366	0,78662	0,84931
687	0,97177	0,84279	0,78438	0,8532
688	0,97105	0,84466	0,78168	0,85192
689	0,97032	0,84451	0,78421	0,84813
690	0,96959	0,84348	0,78628	0,84637
691	0,96887	0,84467	0,78628	0,8413

	A(X)	B(Y)	C(Y)	D(Y)
Long Name	Wavelength	Transmission		
Units	←m	(a.u.)		
Comments		nopol	M	0
692	0,96814	0,84725	0,78714	0,84134
693	0,96742	0,84753	0,79087	0,84565
694	0,9667	0,84662	0,79131	0,84696
695	0,96598	0,84832	0,78782	0,84454
696	0,96526	0,85002	0,7853	0,84007
697	0,96454	0,84932	0,78585	0,83824
698	0,96383	0,84778	0,78591	0,84013
699	0,96311	0,84959	0,786	0,83996
700	0,96239	0,85333	0,78931	0,84004
701	0,96168	0,85504	0,792	0,83897
702	0,96097	0,85297	0,78804	0,83536
703	0,96026	0,85165	0,78737	0,83468
704	0,95954	0,85689	0,78929	0,83428
705	0,95884	0,85934	0,79089	0,83395
706	0,95813	0,85767	0,79271	0,83449
707	0,95742	0,85799	0,79121	0,83349
708	0,95671	0,85862	0,7925	0,83435
709	0,95601	0,86154	0,79556	0,83422
710	0,9553	0,86192	0,79498	0,83189
711	0,9546	0,8621	0,79506	0,8249
712	0,9539	0,86268	0,79672	0,82187
713	0,9532	0,86095	0,79366	0,82079
714	0,95249	0,86207	0,79569	0,82198
715	0,9518	0,86322	0,80041	0,82456
716	0,9511	0,86514	0,80033	0,82361
717	0,9504	0,86761	0,79507	0,81998
