# Supplementary material for: Water-Dispersible Three-Dimensional LC-Nanoresonators
Source: PLoS One. 2014 Aug 25;9(8):e105474. doi: 10.1371/journal.pone.0105474 (PMC4143276; doi:10.1371/journal.pone.0105474)
Supplement: Table S2 — Figure 3B data. (PDF) [file pone.0105474.s005.pdf]

	A(X1)	B(Y1)	C(X2)	D(Y2)	E(X3)	F(Y3)
Long Name	Refractive in	Resonance	Refractive in	Resonance	Refractive in	Resonance
Units	(unit)	←m	(unit)	←m	(unit)	←m
Comments		Water		Ethanol		Isopropanol
1	1,3234	1,2464	1,3546	1,2556	1,368	1,2582
2	1,3234	1,2437	1,3546	1,2532	1,368	1,259
3	1,3234	1,2458	1,3546	1,2552	1,368	1,2573
4	1,3234	1,2474	1,3546	1,2544	1,368	1,2592
