# Supplementary material for: Water-Dispersible Three-Dimensional LC-Nanoresonators
Source: PLoS One. 2014 Aug 25;9(8):e105474. doi: 10.1371/journal.pone.0105474 (PMC4143276; doi:10.1371/journal.pone.0105474)
Supplement: Table S3 — Figure 3C data. (PDF) [file pone.0105474.s006.pdf]

	A(X)	B(Y)	C(Y)	D(Y)	F(Y)
Long Name	Wavelength	Transmission			
Units	←m	(a.u.)			
Comments		0 min	5 min	10 min	20 min
1	1,5004	0,95386	0,99418	0,99608	0,98198
2	1,49867	0,94718	0,96406	0,99351	0,97787
3	1,49693	0,96969	0,97534	0,99597	1,013
4	1,49521	0,95686	0,99384	1,00384	1,03075
5	1,49349	0,97784	0,99971	1,02595	1,0433
6	1,49177	0,99643	1,01451	1,0328	1,03962
7	1,49005	0,95233	1,01647	1,00887	1,01731
8	1,48834	0,93007	1,01873	1,00431	1,03522
9	1,48663	0,95541	1,0078	1,03972	1,04055
10	1,48493	0,98262	1,02935	1,04915	1,03707
11	1,48323	0,95898	0,9897	0,99704	1,00547
12	1,48154	0,94438	0,93436	0,96021	0,98377
13	1,47985	0,95273	0,97434	0,974	0,99173
14	1,47816	0,9473	1,02077	1,03237	1,00389
15	1,47648	0,94721	1,00156	1,03046	0,9901
16	1,4748	0,96096	0,97682	0,98346	0,97616
17	1,47312	0,95589	0,96227	0,98134	0,97912
18	1,47145	0,97187	1,0202	1,03516	1,03957
19	1,46978	0,96899	1,03303	1,03354	1,02814
20	1,46811	0,97285	0,98515	0,98989	0,97099
21	1,46645	0,97011	0,98073	1,00489	0,98066
22	1,4648	0,92732	1,01344	1,03979	0,97692
23	1,46314	0,92748	1,01066	1,02865	0,97411
24	1,46149	0,95985	1,00655	1,02627	0,99766
25	1,45985	0,9838	0,99936	1,0149	1,01901
26	1,45821	0,98413	1,00908	1,01618	1,0127
27	1,45657	0,95662	1,01442	1,0286	0,97859
28	1,45493	0,93919	1,00681	1,00459	0,95123
29	1,4533	0,9296	0,99914	0,96147	0,97423
30	1,45167	0,93567	1,0278	0,98185	0,99539
31	1,45005	0,95923	1,02075	1,0129	1,01238
32	1,44843	0,97398	1,01932	1,04201	1,03955
33	1,44681	0,9483	1,02131	1,03468	1,02541
34	1,4452	0,95243	1,0211	1,02839	1,03913
35	1,44359	0,96112	0,98212	1,01355	1,01871
36	1,44199	0,97211	0,96905	1,0102	1,0012
37	1,44038	0,96693	0,99184	1,00718	1,01
38	1,43878	0,95499	0,99709	0,995	1,03384
39	1,43719	0,96407	1,00246	0,99404	1,06077
40	1,4356	0,95796	0,99187	0,99211	1,01827
41	1,43401	0,92387	0,95832	0,98126	0,96869
42	1,43243	0,8953	0,95396	0,96922	0,96358
43	1,43084	0,87246	0,96934	0,9639	0,97995
44	1,42927	0,8404	0,92371	0,92314	0,97757
45	1,42769	0,88476	0,92361	0,91354	0,98118
46	1,42612	0,9186	0,97272	0,95959	0,98863
47	1,42456	0,90398	1,02782	1,00155	0,98488
48	1,42299	0,89225	1,00511	0,96497	0,94731
49	1,42143	0,88887	0,96379	0,96525	0,93796
50	1,41987	0,91042	0,97139	1,0098	0,98204
51	1,41832	0,89825	0,9831	1,00854	0,99597
52	1,41677	0,87094	0,96194	0,99632	1,00908
53	1,41522	0,88678	0,95987	0,99693	1,00694
54	1,41368	0,88992	0,93148	1,01305	0,97865

	A(X)	B(Y)	C(Y)	D(Y)	F(Y)
Long Name	Wavelength	Transmission			
Units	µm	(a.u.)			
Comments		0 min	5 min	10 min	20 min
55	1,41214	0,86818	0,90453	1,0246	0,98906
56	1,4106	0,89182	0,90809	1,04637	1,02682
57	1,40907	0,90903	0,90475	0,99473	1,00919
58	1,40754	0,89128	0,93038	0,95603	0,9883
59	1,40601	0,87948	0,94604	0,96565	0,94917
60	1,40449	0,86758	0,90763	0,94536	0,89269
61	1,40297	0,85659	0,92373	0,92593	0,91231
62	1,40145	0,85429	0,93022	0,91848	0,92941
63	1,39994	0,85421	0,94936	0,94964	0,93062
64	1,39843	0,8782	0,94488	1,01408	0,95773
65	1,39692	0,9197	0,9146	1,01991	1,00378
66	1,39542	0,94842	0,95591	1,03717	1,03033
67	1,39392	0,92606	0,97936	1,02274	1,01448
68	1,39242	0,91057	0,98933	1,01068	0,99621
69	1,39093	0,8972	0,98448	0,98731	1,00726
70	1,38944	0,85387	0,95507	0,95959	0,99663
71	1,38795	0,8434	0,94106	0,94242	0,95889
72	1,38647	0,87973	0,95981	0,93816	0,95174
73	1,38498	0,87679	1,00113	0,94254	0,96522
74	1,38351	0,92475	1,01311	0,96214	0,9927
75	1,38203	0,96245	0,98908	1,01718	1,02412
76	1,38056	0,8812	0,90759	0,99929	0,97125
77	1,37909	0,80111	0,87386	0,95342	0,91306
78	1,37763	0,80639	0,92261	0,93678	0,94407
79	1,37616	0,87408	0,95487	0,97466	1,00946
80	1,3747	0,87282	0,93508	0,97816	0,97558
81	1,37325	0,84814	0,89135	0,93968	0,95675
82	1,37179	0,86044	0,88603	0,93975	0,96567
83	1,37034	0,84367	0,93217	0,92902	0,91457
84	1,3689	0,84604	0,91995	0,9211	0,90607
85	1,36745	0,85797	0,89405	0,94047	0,91432
86	1,36601	0,83264	0,91955	0,9634	0,92516
87	1,36457	0,81069	0,94955	0,941	0,9394
88	1,36314	0,85414	0,9757	0,96899	0,99058
89	1,36171	0,87171	0,97542	1,01094	1,01163
90	1,36028	0,82438	0,95863	0,96466	0,94033
91	1,35885	0,81492	0,95974	0,94588	0,93579
92	1,35743	0,82348	0,98297	0,98317	1,00122
93	1,35601	0,84399	0,96451	0,98864	1,00594
94	1,35459	0,85339	0,93319	0,93047	0,9547
95	1,35318	0,82486	0,94028	0,9061	0,91656
96	1,35177	0,81611	0,94062	0,92588	0,91225
97	1,35036	0,78949	0,90114	0,90369	0,88884
98	1,34895	0,77643	0,90497	0,8505	0,91429
99	1,34755	0,80161	0,94246	0,87279	0,9305
100	1,34615	0,84058	0,96469	0,93167	0,90415
101	1,34476	0,79535	0,91455	0,93258	0,91065
102	1,34336	0,75641	0,87721	0,9152	0,93936
103	1,34197	0,78172	0,91905	0,94161	0,97435
104	1,34058	0,7977	0,93187	0,95274	0,96398
105	1,3392	0,79859	0,91574	0,93667	0,93469
106	1,33782	0,80617	0,94613	0,93149	0,94009
107	1,33644	0,78446	0,94049	0,93728	0,94401
108	1,33506	0,78524	0,92257	0,9303	0,93042
109	1,33369	0,78116	0,91177	0,93923	0,95098
110	1,33232	0,75362	0,90004	0,93078	0,94107
111	1,33095	0,74399	0,88567	0,9146	0,91205

	A(X)	B(Y)	C(Y)	D(Y)	F(Y)
Long Name	Wavelength	Transmission			
Units	←m	(a.u.)			
Comments		0 min	5 min	10 min	20 min
112	1,32958	0,74451	0,87238	0,90385	0,90307
113	1,32822	0,74504	0,86038	0,89523	0,88882
114	1,32686	0,73821	0,85852	0,90346	0,90662
115	1,32551	0,70916	0,86354	0,88444	0,90689
116	1,32415	0,71439	0,87498	0,89808	0,92698
117	1,3228	0,74208	0,91052	0,94495	0,97752
118	1,32145	0,76357	0,89504	0,98544	0,9741
119	1,32011	0,73409	0,85479	0,94628	0,92819
120	1,31876	0,71206	0,85467	0,92266	0,91198
121	1,31742	0,67888	0,84786	0,90929	0,8808
122	1,31609	0,68223	0,82583	0,88719	0,8677
123	1,31475	0,70451	0,82351	0,89638	0,89156
124	1,31342	0,71446	0,8487	0,90322	0,92258
125	1,31209	0,67043	0,87855	0,87947	0,91154
126	1,31076	0,6526	0,8514	0,87099	0,88448
127	1,30944	0,66171	0,82871	0,87666	0,87048
128	1,30812	0,65552	0,85313	0,89912	0,90352
129	1,3068	0,67695	0,87579	0,89839	0,92001
130	1,30548	0,70871	0,88454	0,86496	0,91245
131	1,30417	0,69214	0,86144	0,85528	0,90245
132	1,30286	0,63811	0,8502	0,88454	0,90287
133	1,30155	0,56223	0,83832	0,84717	0,91018
134	1,30025	0,58764	0,80824	0,7659	0,88681
135	1,29894	0,641	0,83119	0,7745	0,89174
136	1,29764	0,63138	0,86765	0,8505	0,9244
137	1,29635	0,60873	0,85808	0,86539	0,90741
138	1,29505	0,60545	0,84573	0,85166	0,87383
139	1,29376	0,62669	0,82628	0,88024	0,88528
140	1,29247	0,61146	0,78211	0,87689	0,90259
141	1,29118	0,58934	0,7838	0,83712	0,91128
142	1,2899	0,61504	0,82662	0,81436	0,89257
143	1,28861	0,60832	0,81294	0,77327	0,82578
144	1,28733	0,58171	0,82403	0,7664	0,81906
145	1,28606	0,60491	0,83262	0,81472	0,84459
146	1,28478	0,56853	0,78405	0,8533	0,83305
147	1,28351	0,51022	0,73582	0,85033	0,81201
148	1,28224	0,53963	0,72369	0,83472	0,80374
149	1,28097	0,59957	0,74077	0,83711	0,8224
150	1,27971	0,58502	0,76649	0,82244	0,84272
151	1,27845	0,57786	0,78732	0,8108	0,8468
152	1,27719	0,57123	0,80298	0,80627	0,83091
153	1,27593	0,56217	0,77975	0,82294	0,8199
154	1,27468	0,56505	0,76852	0,84476	0,82563
155	1,27342	0,56529	0,81778	0,84585	0,84213
156	1,27217	0,57112	0,83721	0,86496	0,87392
157	1,27093	0,52014	0,79328	0,85244	0,86634
158	1,26968	0,49636	0,75681	0,80056	0,82674
159	1,26844	0,51668	0,73308	0,77014	0,80999
160	1,2672	0,53829	0,72882	0,76162	0,81834
161	1,26596	0,50473	0,74327	0,76392	0,81345
162	1,26473	0,49616	0,73712	0,77041	0,80755
163	1,26349	0,52265	0,76312	0,7912	0,78414
164	1,26226	0,52708	0,78716	0,78844	0,80907
165	1,26104	0,54809	0,72762	0,7907	0,80681
166	1,25981	0,50974	0,7471	0,79157	0,80418
167	1,25859	0,48318	0,75926	0,77628	0,80545
168	1,25737	0,52009	0,74005	0,7729	0,80565
169	1,25615	0,5432	0,71186	0,74634	0,81771

	A(X)	B(Y)	C(Y)	D(Y)	F(Y)
Long Name	Wavelength	Transmission			
Units	←m	(a.u.)			
Comments		0 min	5 min	10 min	20 min
170	1,25493	0,54813	0,71739	0,74285	0,82354
171	1,25372	0,53153	0,73133	0,78277	0,80587
172	1,25251	0,51225	0,74064	0,78078	0,82501
173	1,2513	0,50868	0,69435	0,74806	0,80592
174	1,25009	0,48994	0,69974	0,72925	0,79073
175	1,24889	0,45284	0,70253	0,77014	0,79673
176	1,24769	0,46372	0,67293	0,78489	0,73204
177	1,24649	0,50625	0,66851	0,73327	0,70075
178	1,24529	0,51719	0,68298	0,67921	0,7098
179	1,24409	0,50703	0,69063	0,67902	0,70687
180	1,2429	0,51888	0,69863	0,7457	0,76107
181	1,24171	0,48981	0,68061	0,74679	0,75786
182	1,24052	0,49823	0,68263	0,7642	0,76624
183	1,23934	0,52259	0,69339	0,75531	0,77566
184	1,23815	0,50539	0,66495	0,69452	0,75983
185	1,23697	0,50082	0,63963	0,70227	0,75807
186	1,23579	0,50673	0,64944	0,74427	0,78209
187	1,23461	0,50348	0,63845	0,74656	0,75518
188	1,23344	0,47657	0,64555	0,71642	0,71402
189	1,23227	0,46443	0,6293	0,73526	0,72205
190	1,2311	0,49417	0,66391	0,72655	0,74605
191	1,22993	0,4903	0,69502	0,66797	0,74854
192	1,22876	0,48679	0,66186	0,69999	0,73894
193	1,2276	0,51331	0,62211	0,73201	0,72984
194	1,22644	0,51919	0,65592	0,7279	0,74642
195	1,22528	0,47642	0,64881	0,67916	0,72624
196	1,22412	0,48056	0,63717	0,64898	0,72905
197	1,22297	0,50191	0,65809	0,69774	0,74111
198	1,22181	0,47107	0,6461	0,66876	0,70576
199	1,22066	0,47853	0,64699	0,62554	0,69058
200	1,21952	0,52741	0,69188	0,62914	0,70991
201	1,21837	0,51628	0,68358	0,63277	0,70838
202	1,21723	0,48406	0,68652	0,70532	0,71858
203	1,21608	0,44308	0,6369	0,73271	0,77755
204	1,21494	0,42733	0,55494	0,67173	0,76828
205	1,21381	0,43608	0,54967	0,66278	0,71152
206	1,21267	0,49054	0,58284	0,65285	0,681
207	1,21154	0,50931	0,61522	0,64497	0,68463
208	1,21041	0,50811	0,60243	0,65348	0,68089
209	1,20928	0,50801	0,62915	0,64575	0,70373
210	1,20815	0,48438	0,66393	0,65212	0,73065
211	1,20703	0,50295	0,64212	0,69211	0,7498
212	1,2059	0,5222	0,59918	0,69133	0,75177
213	1,20478	0,50602	0,5771	0,65059	0,71528
214	1,20366	0,52728	0,58704	0,66188	0,70034
215	1,20255	0,53488	0,64019	0,67692	0,67852
216	1,20143	0,49479	0,63352	0,65983	0,67663
217	1,20032	0,47666	0,62618	0,60749	0,69696
218	1,19921	0,50868	0,64244	0,61408	0,76802
219	1,1981	0,52874	0,61465	0,6636	0,77187
220	1,19699	0,52778	0,60344	0,64133	0,68913
221	1,19589	0,55591	0,63783	0,6414	0,69108
222	1,19479	0,583	0,63362	0,62025	0,73818
223	1,19369	0,52564	0,6172	0,64143	0,72143
224	1,19259	0,50082	0,62201	0,70391	0,70337
225	1,19149	0,53835	0,6017	0,64982	0,69791
226	1,1904	0,48182	0,62432	0,57921	0,67098
227	1,18931	0,50999	0,67537	0,60545	0,66305

	A(X)	B(Y)	C(Y)	D(Y)	F(Y)
Long Name	Wavelength	Transmission			
Units	‹m	(a.u.)			
Comments		0 min	5 min	10 min	20 min
228	1,18822	0,52993	0,69137	0,59354	0,62545
229	1,18713	0,49564	0,64622	0,58768	0,60888
230	1,18604	0,54831	0,63477	0,6709	0,63174
231	1,18496	0,62144	0,63907	0,67951	0,71442
232	1,18388	0,69992	0,64276	0,68115	0,81291
233	1,1828	0,69655	0,64322	0,70786	0,78601
234	1,18172	0,57712	0,63173	0,69771	0,71606
235	1,18064	0,52209	0,63028	0,71507	0,69598
236	1,17957	0,55467	0,67642	0,71987	0,72318
237	1,1785	0,56324	0,68298	0,6662	0,73243
238	1,17743	0,57834	0,6363	0,58762	0,76232
239	1,17636	0,6125	0,58738	0,59924	0,80089
240	1,17529	0,58284	0,5986	0,6582	0,76727
241	1,17423	0,55693	0,62695	0,61492	0,71853
242	1,17316	0,60963	0,61065	0,59388	0,66938
243	1,1721	0,63482	0,62708	0,62815	0,64614
244	1,17104	0,59061	0,64797	0,66138	0,70028
245	1,16999	0,59844	0,67144	0,68221	0,75987
246	1,16893	0,57205	0,70214	0,66878	0,74901
247	1,16788	0,5587	0,6749	0,66624	0,70022
248	1,16683	0,62691	0,66427	0,72155	0,70812
249	1,16578	0,62498	0,65056	0,69698	0,69795
250	1,16473	0,57245	0,62268	0,65307	0,68408
251	1,16369	0,57884	0,65372	0,66426	0,73333
252	1,16264	0,63032	0,71066	0,72615	0,79749
253	1,1616	0,64568	0,69132	0,73818	0,75807
254	1,16056	0,63718	0,65667	0,67828	0,75318
255	1,15952	0,62625	0,64872	0,64667	0,77075
256	1,15849	0,61019	0,62897	0,68799	0,73794
257	1,15745	0,61777	0,63101	0,69757	0,74969
258	1,15642	0,6226	0,63682	0,6941	0,77257
259	1,15539	0,63891	0,67967	0,69552	0,73843
260	1,15436	0,67721	0,7037	0,70066	0,73415
261	1,15333	0,66737	0,70579	0,69665	0,74139
262	1,15231	0,61482	0,67974	0,66432	0,7106
263	1,15128	0,62308	0,66871	0,67492	0,72076
264	1,15026	0,65636	0,69053	0,68374	0,79162
265	1,14924	0,6275	0,72197	0,65971	0,77521
266	1,14822	0,5834	0,6962	0,64397	0,70758
267	1,14721	0,62925	0,72187	0,69342	0,70228
268	1,14619	0,67818	0,72833	0,71252	0,70114
269	1,14518	0,69835	0,74323	0,71984	0,69706
270	1,14417	0,72322	0,72128	0,73261	0,73904
271	1,14316	0,68394	0,70063	0,70928	0,73631
272	1,14215	0,67323	0,71033	0,71844	0,75886
273	1,14115	0,73695	0,70359	0,76874	0,79965
274	1,14015	0,71326	0,73289	0,75749	0,77729
275	1,13914	0,62489	0,71992	0,72582	0,74993
276	1,13814	0,61823	0,70192	0,73383	0,75757
277	1,13715	0,68025	0,65896	0,74298	0,76031
278	1,13615	0,70261	0,64401	0,73	0,72577
279	1,13515	0,69014	0,67753	0,67486	0,70771
280	1,13416	0,65797	0,69755	0,61449	0,72983
281	1,13317	0,58045	0,66517	0,64802	0,70846
282	1,13218	0,58066	0,67736	0,71992	0,68303
283	1,13119	0,66239	0,74149	0,77028	0,72015
284	1,13021	0,66486	0,7004	0,75941	0,73842
285	1,12922	0,63896	0,7012	0,73022	0,75916

	A(X)	B(Y)	C(Y)	D(Y)	F(Y)
Long Name	Wavelength	Transmission			
Units	←m	(a.u.)			
Comments		0 min	5 min	10 min	20 min
286	1,12824	0,70311	0,79196	0,72879	0,77311
287	1,12726	0,74358	0,78116	0,72922	0,80126
288	1,12628	0,76367	0,74178	0,75127	0,79768
289	1,1253	0,76898	0,71951	0,77012	0,78747
290	1,12432	0,71805	0,69464	0,73329	0,80681
291	1,12335	0,67336	0,69546	0,72188	0,84752
292	1,12238	0,69905	0,73492	0,72654	0,87223
293	1,12141	0,73825	0,7363	0,713	0,79528
294	1,12044	0,70861	0,72093	0,73083	0,73791
295	1,11947	0,67432	0,74177	0,77131	0,77964
296	1,1185	0,71839	0,71666	0,76484	0,83458
297	1,11754	0,75622	0,70609	0,72144	0,80748
298	1,11658	0,72004	0,698	0,70553	0,76691
299	1,11562	0,64612	0,65802	0,68048	0,7067
300	1,11466	0,62596	0,74194	0,69055	0,72151
301	1,1137	0,6539	0,80953	0,72876	0,77942
302	1,11274	0,69043	0,79727	0,74373	0,8088
303	1,11179	0,75862	0,82257	0,79954	0,82331
304	1,11084	0,72529	0,83477	0,82653	0,84822
305	1,10988	0,70665	0,79755	0,77476	0,83015
306	1,10894	0,69745	0,73698	0,75427	0,79463
307	1,10799	0,67651	0,698	0,7406	0,80003
308	1,10704	0,70477	0,70657	0,73358	0,8121
309	1,1061	0,73347	0,68566	0,72981	0,79914
310	1,10515	0,74341	0,66503	0,76808	0,75585
311	1,10421	0,73327	0,6686	0,79447	0,71456
312	1,10327	0,74524	0,66364	0,77567	0,74477
313	1,10233	0,71852	0,66823	0,74852	0,74988
314	1,1014	0,67675	0,7127	0,7365	0,72183
315	1,10046	0,6612	0,80261	0,7509	0,80161
316	1,09953	0,65776	0,78081	0,73141	0,86868
317	1,0986	0,66858	0,70675	0,73163	0,82658
318	1,09767	0,67668	0,69975	0,77099	0,79408
319	1,09674	0,69553	0,69746	0,77252	0,76281
320	1,09581	0,6474	0,66732	0,71036	0,69461
321	1,09489	0,63286	0,69857	0,69915	0,69434
322	1,09396	0,67819	0,76628	0,74189	0,75431
323	1,09304	0,68052	0,76683	0,77837	0,74038
324	1,09212	0,67286	0,6962	0,75061	0,70294
325	1,0912	0,72397	0,71831	0,75476	0,75556
326	1,09028	0,71799	0,73391	0,75698	0,7997
327	1,08937	0,68613	0,68056	0,71412	0,76838
328	1,08845	0,66087	0,6785	0,70448	0,75972
329	1,08754	0,65471	0,76828	0,75602	0,77747
330	1,08663	0,64448	0,74194	0,79338	0,71127
331	1,08572	0,71603	0,69637	0,7611	0,72534
332	1,08481	0,80657	0,77864	0,79619	0,85397
333	1,0839	0,76862	0,77207	0,77453	0,81796
334	1,083	0,72102	0,71369	0,73366	0,74609
335	1,08209	0,70449	0,70559	0,77543	0,7202
336	1,08119	0,67545	0,73841	0,82871	0,77208
337	1,08029	0,67397	0,78694	0,82777	0,78258
338	1,07939	0,68556	0,79186	0,7887	0,69601
339	1,07849	0,74329	0,73111	0,74766	0,67245
340	1,07759	0,7752	0,69416	0,78155	0,73268
341	1,0767	0,81985	0,741	0,82402	0,80424
342	1,07581	0,73629	0,7844	0,79929	0,82863
343	1,07491	0,62206	0,6961	0,76825	0,7673

	A(X)	B(Y)	C(Y)	D(Y)	F(Y)
Long Name	Wavelength	Transmission			
Units	←m	(a.u.)			
Comments		0 min	5 min	10 min	20 min
344	1,07402	0,62757	0,72879	0,73246	0,76959
345	1,07313	0,659	0,74152	0,72504	0,77734
346	1,07225	0,73702	0,74491	0,78521	0,77789
347	1,07136	0,76887	0,81437	0,80685	0,78408
348	1,07048	0,74204	0,80754	0,79211	0,78674
349	1,06959	0,76347	0,77298	0,86023	0,83323
350	1,06871	0,79172	0,77493	0,85556	0,83831
351	1,06783	0,76081	0,76043	0,74021	0,77117
352	1,06695	0,68768	0,74542	0,68715	0,70743
353	1,06607	0,66637	0,74914	0,72151	0,72838
354	1,0652	0,67853	0,74278	0,70975	0,72047
355	1,06432	0,69277	0,71681	0,65376	0,69557
356	1,06345	0,71633	0,73971	0,62603	0,73559
357	1,06258	0,69368	0,72312	0,62434	0,80736
358	1,06171	0,66379	0,69864	0,67838	0,77284
359	1,06084	0,66078	0,72453	0,75581	0,75038
360	1,05997	0,67962	0,69191	0,81068	0,8053
361	1,05911	0,67861	0,72465	0,83103	0,80299
362	1,05824	0,6698	0,78858	0,83599	0,73879
363	1,05738	0,69458	0,77775	0,80888	0,72544
364	1,05652	0,74358	0,79478	0,79601	0,81223
365	1,05566	0,70193	0,79963	0,8273	0,8851
366	1,0548	0,72754	0,79064	0,86897	0,86436
367	1,05394	0,78592	0,76292	0,8587	0,81264
368	1,05308	0,69699	0,70063	0,84858	0,74246
369	1,05223	0,65402	0,70698	0,83935	0,76694
370	1,05138	0,7313	0,75724	0,79967	0,81195
371	1,05052	0,70087	0,72124	0,71912	0,79876
372	1,04967	0,70046	0,73522	0,73204	0,8269
373	1,04882	0,7297	0,72372	0,7638	0,78245
374	1,04798	0,75069	0,72406	0,74484	0,70636
375	1,04713	0,71892	0,78943	0,74542	0,68512
376	1,04628	0,65016	0,78751	0,72952	0,74715
377	1,04544	0,66222	0,74371	0,72781	0,79997
378	1,0446	0,731	0,80247	0,78097	0,87542
379	1,04376	0,72585	0,85577	0,77979	0,85891
380	1,04292	0,74533	0,85065	0,82558	0,87398
381	1,04208	0,74871	0,81044	0,8328	0,91646
382	1,04124	0,63648	0,73545	0,73627	0,8391
383	1,04041	0,63623	0,71895	0,75253	0,80054
384	1,03957	0,69685	0,71891	0,74239	0,77531
385	1,03874	0,69992	0,68586	0,70852	0,71867
386	1,03791	0,65763	0,69601	0,78486	0,70309
387	1,03708	0,65011	0,78412	0,8109	0,74334
388	1,03625	0,661	0,7951	0,79172	0,79321
389	1,03542	0,70895	0,79059	0,74568	0,78531
390	1,03459	0,71619	0,80067	0,74403	0,76306
391	1,03377	0,74806	0,83996	0,78756	0,74214
392	1,03294	0,72044	0,81073	0,80221	0,73133
393	1,03212	0,70573	0,77011	0,70985	0,721
394	1,0313	0,71435	0,71005	0,65654	0,73046
395	1,03048	0,66438	0,69665	0,71397	0,77767
396	1,02966	0,6719	0,70357	0,82544	0,85707
397	1,02885	0,72273	0,72358	0,89629	0,84809
398	1,02803	0,70983	0,77024	0,81749	0,69528
399	1,02721	0,74882	0,80022	0,76564	0,70918
400	1,0264	0,87759	0,81267	0,89899	0,82354
401	1,02559	0,93199	0,82873	0,88967	0,81744

	A(X)	B(Y)	C(Y)	D(Y)	F(Y)
Long Name	Wavelength	Transmission			
Units	←m	(a.u.)			
Comments		0 min	5 min	10 min	20 min
402	1,02478	0,82767	0,88383	0,86686	0,85589
403	1,02397	0,69791	0,8816	0,90066	0,87874
404	1,02316	0,78523	0,89917	0,89134	0,90981
405	1,02235	0,80209	0,9123	0,82909	0,89765
406	1,02155	0,72135	0,81895	0,73207	0,82727
407	1,02074	0,68902	0,78334	0,77878	0,86661
408	1,01994	0,7076	0,81258	0,83596	0,84442
409	1,01914	0,76261	0,81552	0,77743	0,78986
410	1,01834	0,71973	0,71116	0,72145	0,71598
411	1,01754	0,76936	0,70863	0,83422	0,69683
412	1,01674	0,77446	0,81101	0,88926	0,76001
413	1,01594	0,73902	0,78287	0,80911	0,75229
414	1,01515	0,68545	0,71022	0,76596	0,70133
415	1,01435	0,66041	0,69255	0,68949	0,74482
416	1,01356	0,6894	0,69862	0,69513	0,8127
417	1,01277	0,69979	0,71243	0,76137	0,74366
418	1,01198	0,68596	0,73283	0,75917	0,75828
419	1,01119	0,69564	0,73158	0,72413	0,7705
420	1,0104	0,67737	0,75958	0,72699	0,73954
421	1,00961	0,67003	0,80314	0,75324	0,73167
422	1,00883	0,76477	0,86648	0,75717	0,7623
423	1,00804	0,84059	0,90552	0,79717	0,78624
424	1,00726	0,88231	0,99376	0,8697	0,91767
425	1,00648	0,799	0,93913	0,78666	0,94298
426	1,0057	0,74899	0,90784	0,77427	0,85985
427	1,00492	0,78744	0,8782	0,80817	0,77538
428	1,00414	0,72879	0,8642	0,79502	0,71707
429	1,00336	0,72818	0,81882	0,82943	0,74592
430	1,00259	0,77682	0,76278	0,92383	0,91641
431	1,00181	0,74396	0,78764	0,96827	0,9784
432	1,00104	0,66378	0,73265	0,91665	0,81134
433	1,00027	0,69035	0,7436	0,82464	0,76546
