# Supplementary material for: Water-Dispersible Three-Dimensional LC-Nanoresonators
Source: PLoS One. 2014 Aug 25;9(8):e105474. doi: 10.1371/journal.pone.0105474 (PMC4143276; doi:10.1371/journal.pone.0105474)
Supplement: Table S4 — Figure 3D data. (PDF) [file pone.0105474.s007.pdf]

	A(X)	B(Y)	C(xEr±)
Long Name	Time	Max (Absorp	Errorx
Units	(min)	(unit)	min
Comments			
1	1	0,5182	1
2	6	0,3428	1
3	11	0,3243	1
4	16	0,2908	1
5	21	0,2705	1
6	26	0,2598	1
7	31	0,2546	1
