# Supplementary material for: Water-Dispersible Three-Dimensional LC-Nanoresonators
Source: PLoS One. 2014 Aug 25;9(8):e105474. doi: 10.1371/journal.pone.0105474 (PMC4143276; doi:10.1371/journal.pone.0105474)
Supplement: Table S5 — Figure 4 data. (PDF) [file pone.0105474.s008.pdf]

	A(X)	B(Y)	D(Y)
Long Name	Wavelength	Reflectivity	
Units	←m	(a.u.)	
Comments		water	DMSO
1	0,96526	1,02976	1,0034
2	0,96598	0,98034	0,99972
3	0,9667	0,98034	0,95575
4	0,96742	0,97138	0,94253
5	0,96814	0,97138	0,98063
6	0,96887	0,97114	0,98093
7	0,96959	0,97114	0,97659
8	0,97032	0,98214	1,0039
9	0,97105	0,98214	0,98552
10	0,97177	1,01061	0,97431
11	0,9725	1,01061	0,9748
12	0,97323	0,99365	0,95136
13	0,97396	0,99365	0,94102
14	0,9747	0,98965	0,96958
15	0,97543	0,98965	1,00698
16	0,97616	0,97079	0,99504
17	0,9769	0,97079	0,99532
18	0,97764	0,99254	0,996
19	0,97837	0,99254	0,9821
20	0,97911	0,9896	0,99317
21	0,97985	0,9896	0,99594
22	0,98059	0,98469	0,98748
23	0,98134	0,98469	0,95862
24	0,98208	0,9829	0,95677
25	0,98282	0,9829	0,96549
26	0,98357	0,96492	0,96502
27	0,98432	0,96492	0,97849
28	0,98506	0,98162	0,9927
29	0,98581	0,98162	0,97271
30	0,98656	0,98615	0,97139
31	0,98732	0,98615	0,97845
32	0,98807	0,97255	0,98519
33	0,98882	0,97255	0,96272
34	0,98958	0,98833	0,93287
35	0,99033	0,98833	0,94797
36	0,99109	0,99212	0,96923
37	0,99185	0,99212	0,96082
38	0,99261	0,96956	0,95954
39	0,99337	0,96956	0,99272
40	0,99413	0,97473	1,01683
41	0,99489	0,97473	0,99791
42	0,99566	0,95532	0,96717
43	0,99642	0,95532	0,96146
44	0,99719	0,97422	0,96144
45	0,99796	0,97422	0,97228
46	0,99873	0,99012	1,00092
47	0,9995	0,99012	0,99098
48	1,00027	0,98537	0,9552
49	1,00104	0,98537	0,96684
50	1,00181	0,99702	0,98438
51	1,00259	0,99702	0,97977
52	1,00336	0,98628	0,98451
53	1,00414	0,98628	0,98456
54	1,00492	0,99792	0,9669

	A(X)	B(Y)	D(Y)
Long Name	Wavelength	Reflectivity	
Units	←m	(a.u.)	
Comments		water	DMSO
55	1,0057	0,99792	0,94561
56	1,00648	0,99932	0,92983
57	1,00726	0,99932	0,95063
58	1,00804	0,98545	0,95481
59	1,00883	0,98545	0,95336
60	1,00961	0,99927	0,96497
61	1,0104	0,99927	1,00293
62	1,01119	1,00187	0,9953
63	1,01198	1,00187	0,96753
64	1,01277	0,98175	0,96587
65	1,01356	0,98175	0,97465
66	1,01435	0,96725	0,98193
67	1,01515	0,96725	0,96316
68	1,01594	0,97513	0,94636
69	1,01674	0,97513	0,96221
70	1,01754	0,97757	0,97272
71	1,01834	0,97757	0,95654
72	1,01914	0,98944	0,952
73	1,01994	0,98944	0,96216
74	1,02074	1,01214	0,96758
75	1,02155	1,01214	0,9761
76	1,02235	1,00575	0,96904
77	1,02316	1,00575	0,97242
78	1,02397	1,00603	0,97368
79	1,02478	1,00603	0,98758
80	1,02559	0,98583	1,00222
81	1,0264	0,98583	0,96404
82	1,02721	0,99153	0,96351
83	1,02803	0,99153	0,98795
84	1,02885	0,98194	0,98821
85	1,02966	0,98194	0,97165
86	1,03048	0,99457	0,96338
87	1,0313	0,99457	0,95955
88	1,03212	0,98132	0,96036
89	1,03294	0,98132	0,96691
90	1,03377	0,98717	0,97734
91	1,03459	0,98717	0,96535
92	1,03542	0,99028	0,95947
93	1,03625	0,99028	0,9714
94	1,03708	0,98008	0,97806
95	1,03791	0,98008	0,98235
96	1,03874	0,99491	0,97829
97	1,03957	0,99491	0,97985
98	1,04041	0,98323	0,98842
99	1,04124	0,98323	0,99088
100	1,04208	0,98671	0,99639
101	1,04292	0,98671	0,98182
102	1,04376	0,99522	0,97497
103	1,0446	0,99522	0,97317
104	1,04544	0,97477	0,96228
105	1,04628	0,97477	0,95704
106	1,04713	0,99168	0,96067
107	1,04798	0,99168	0,96036
108	1,04882	0,98464	0,967
109	1,04967	0,98464	0,98861
110	1,05052	0,98816	0,98944
111	1,05138	0,98816	0,98491

	A(X)	B(Y)	D(Y)
Long Name	Wavelength	Reflectivity	
Units	←m	(a.u.)	
Comments		water	DMSO
112	1,05223	0,96226	0,97411
113	1,05308	0,96226	0,97316
114	1,05394	0,97054	0,9736
115	1,0548	0,97054	0,95895
116	1,05566	1,00108	0,9308
117	1,05652	1,00108	0,93303
118	1,05738	0,99961	0,9666
119	1,05824	0,99961	0,96969
120	1,05911	0,99971	0,9592
121	1,05997	0,99971	0,95173
122	1,06084	1,00515	0,94504
123	1,06171	1,00515	0,94696
124	1,06258	0,9827	0,94838
125	1,06345	0,9827	0,95981
126	1,06432	0,98081	0,95096
127	1,0652	0,98081	0,94153
128	1,06607	0,9704	0,94531
129	1,06695	0,9704	0,95932
130	1,06783	0,99397	0,98204
131	1,06871	0,99397	0,98494
132	1,06959	0,9893	0,98653
133	1,07048	0,9893	0,98219
134	1,07136	0,99281	0,9629
135	1,07225	0,99281	0,9578
136	1,07313	0,9868	0,96676
137	1,07402	0,9868	0,96026
138	1,07491	0,99607	0,95789
139	1,07581	0,99607	0,95284
140	1,0767	0,9801	0,96504
141	1,07759	0,9801	0,96862
142	1,07849	0,98358	0,96833
143	1,07939	0,98358	0,96978
144	1,08029	1,00041	0,95483
145	1,08119	1,00041	0,9655
146	1,08209	1,00235	0,96964
147	1,083	1,00235	0,95703
148	1,0839	0,98735	0,96263
149	1,08481	0,98735	0,96839
150	1,08572	0,98847	0,9829
151	1,08663	0,98847	0,99397
152	1,08754	1,00027	0,99846
153	1,08845	1,00027	0,99033
154	1,08937	1,01499	0,97506
155	1,09028	1,01499	0,95323
156	1,0912	1,00124	0,9633
157	1,09212	1,00124	0,9718
158	1,09304	0,99315	0,9489
159	1,09396	0,99315	0,94639
160	1,09489	0,9984	0,96984
161	1,09581	0,9984	0,99275
162	1,09674	0,99373	1,00059
163	1,09767	0,99373	0,98782
164	1,0986	0,99193	0,98338
165	1,09953	0,99193	0,97452
166	1,10046	0,99504	0,97314
167	1,1014	0,99504	0,98735
168	1,10233	0,99495	0,98163
169	1,10327	0,99495	0,96689

	A(X)	B(Y)	D(Y)
Long Name	Wavelength	Reflectivity	
Units	←m	(a.u.)	
Comments		water	DMSO
170	1,10421	0,99386	0,97295
171	1,10515	0,99386	0,97639
172	1,1061	0,98892	0,97849
173	1,10704	0,98892	0,98277
174	1,10799	0,98338	0,98198
175	1,10894	0,98338	0,98206
176	1,10988	0,99063	0,97106
177	1,11084	0,99063	0,9705
178	1,11179	0,99242	0,97257
179	1,11274	0,99242	0,96942
180	1,1137	0,99949	0,97051
181	1,11466	0,99949	0,9708
182	1,11562	0,99862	0,9688
183	1,11658	0,99862	0,96696
184	1,11754	1,00195	0,97029
185	1,1185	1,00195	0,97751
186	1,11947	1,00983	0,97017
187	1,12044	1,00983	0,95551
188	1,12141	0,99159	0,9559
189	1,12238	0,99159	0,96177
190	1,12335	0,98939	0,96489
191	1,12432	0,98939	0,96503
192	1,1253	1,00394	0,97414
193	1,12628	1,00394	0,98591
194	1,12726	1,00116	0,97687
195	1,12824	1,00116	0,97194
196	1,12922	1,00017	0,97874
197	1,13021	1,00017	0,96961
198	1,13119	1,00605	0,95826
199	1,13218	1,00605	0,96588
200	1,13317	1,01251	0,95884
201	1,13416	1,01251	0,9561
202	1,13515	1,00236	0,96284
203	1,13615	1,00236	0,97297
204	1,13715	0,99547	0,98526
205	1,13814	0,99547	0,98427
206	1,13914	0,99879	0,97378
207	1,14015	0,99879	0,9755
208	1,14115	1,00059	0,96474
209	1,14215	1,00059	0,95038
210	1,14316	0,99826	0,9519
211	1,14417	0,99826	0,97205
212	1,14518	1,00641	0,98946
213	1,14619	1,00641	0,99004
214	1,14721	1,00999	0,98785
215	1,14822	1,00999	0,98102
216	1,14924	1,00376	0,96803
217	1,15026	1,00376	0,9642
218	1,15128	1,00102	0,96607
219	1,15231	1,00102	0,97848
220	1,15333	1,00218	0,98373
221	1,15436	1,00218	0,97654
222	1,15539	1,00362	0,98137
223	1,15642	1,00362	0,98166
224	1,15745	1,00065	0,97181
225	1,15849	1,00065	0,97295
226	1,15952	1,00642	0,98877
227	1,16056	1,00642	0,98879

	A(X)	B(Y)	D(Y)
Long Name	Wavelength	Reflectivity	
Units	←m	(a.u.)	
Comments		water	DMSO
228	1,1616	1,00293	0,97078
229	1,16264	1,00293	0,96151
230	1,16369	1,00701	0,96492
231	1,16473	1,00701	0,9781
232	1,16578	1,00714	0,98446
233	1,16683	1,00714	0,97254
234	1,16788	1,0076	0,96247
235	1,16893	1,0076	0,95766
236	1,16999	1,00773	0,96045
237	1,17104	1,00773	0,96731
238	1,1721	1,00381	0,97395
239	1,17316	1,00381	0,97938
240	1,17423	1,00596	0,96491
241	1,17529	1,00596	0,96005
242	1,17636	1,00219	0,96967
243	1,17743	1,00219	0,98172
244	1,1785	1,00555	0,98353
245	1,17957	1,00555	0,98074
246	1,18064	1,00139	0,98064
247	1,18172	1,00139	0,97222
248	1,1828	1,00529	0,97087
249	1,18388	1,00529	0,95999
250	1,18496	1,0101	0,95048
251	1,18604	1,0101	0,95133
252	1,18713	1,00507	0,96552
253	1,18822	1,00507	0,97704
254	1,18931	0,99729	0,97526
255	1,1904	0,99729	0,97901
256	1,19149	1,01245	0,9733
257	1,19259	1,01245	0,96915
258	1,19369	0,99854	0,96499
259	1,19479	0,99854	0,96138
260	1,19589	1,00936	0,96394
261	1,19699	1,00936	0,96256
262	1,1981	1,00844	0,96944
263	1,19921	1,00844	0,98856
264	1,20032	1,00163	0,99154
265	1,20143	1,00163	0,98314
266	1,20255	1,0011	0,97932
267	1,20366	1,0011	0,97404
268	1,20478	1,00013	0,97262
269	1,2059	1,00013	0,97041
270	1,20703	1,00453	0,96207
271	1,20815	1,00453	0,96247
272	1,20928	1,00862	0,96352
273	1,21041	1,00862	0,96635
274	1,21154	1,01404	0,96329
275	1,21267	1,01404	0,96997
276	1,21381	1,01625	0,98224
277	1,21494	1,01625	0,97643
278	1,21608	1,00772	0,96297
279	1,21723	1,00772	0,95788
280	1,21837	1,01532	0,96333
281	1,21952	1,01532	0,96102
282	1,22066	1,00611	0,95701
283	1,22181	1,00611	0,96508
284	1,22297	1,00302	0,98003
285	1,22412	1,00302	0,97903

	A(X)	B(Y)	D(Y)
Long Name	Wavelength	Reflectivity	
Units	←m	(a.u.)	
Comments		water	DMSO
286	1,22528	1,00469	0,97649
287	1,22644	1,00469	0,97235
288	1,2276	1,00838	0,96549
289	1,22876	1,00838	0,96744
290	1,22993	1,00896	0,97319
291	1,2311	1,00896	0,96824
292	1,23227	1,00954	0,97232
293	1,23344	1,00954	0,97422
294	1,23461	1,00711	0,97284
295	1,23579	1,00711	0,97285
296	1,23697	1,00962	0,9701
297	1,23815	1,00962	0,96463
298	1,23934	1,01089	0,97165
299	1,24052	1,01089	0,97374
300	1,24171	1,01297	0,97035
301	1,2429	1,01297	0,96833
302	1,24409	1,00207	0,96614
303	1,24529	1,00207	0,97467
304	1,24649	1,00559	0,97946
305	1,24769	1,00559	0,97099
306	1,24889	1,01485	0,96601
307	1,25009	1,01485	0,97459
308	1,2513	1,01093	0,96669
309	1,25251	1,01093	0,96205
310	1,25372	1,0025	0,96486
311	1,25493	1,0025	0,96355
312	1,25615	1,00941	0,96886
313	1,25737	1,00941	0,97436
314	1,25859	1,00884	0,97575
315	1,25981	1,00884	0,98137
316	1,26104	1,01339	0,97978
317	1,26226	1,01339	0,96985
318	1,26349	1,00784	0,97141
319	1,26473	1,00784	0,97394
320	1,26596	1,01155	0,97189
321	1,2672	1,01155	0,97218
322	1,26844	1,0138	0,96524
323	1,26968	1,0138	0,96516
324	1,27093	1,00775	0,97491
325	1,27217	1,00775	0,98493
326	1,27342	1,0071	0,98218
327	1,27468	1,0071	0,97937
328	1,27593	1,00882	0,97932
329	1,27719	1,00882	0,96921
330	1,27845	1,00859	0,96982
331	1,27971	1,00859	0,96894
332	1,28097	1,0147	0,96486
333	1,28224	1,0147	0,97187
334	1,28351	1,00984	0,97484
335	1,28478	1,00984	0,97463
336	1,28606	1,01198	0,98103
337	1,28733	1,01198	0,98114
338	1,28861	1,01338	0,96686
339	1,2899	1,01338	0,96542
340	1,29118	1,01201	0,96736
341	1,29247	1,01201	0,97203
342	1,29376	1,01511	0,97156
343	1,29505	1,01511	0,9688

	A(X)	B(Y)	D(Y)
Long Name	Wavelength	Reflectivity	
Units	←m	(a.u.)	
Comments		water	DMSO
344	1,29635	1,01076	0,97468
345	1,29764	1,01076	0,98039
346	1,29894	1,00667	0,98262
347	1,30025	1,00667	0,98006
348	1,30155	1,01249	0,98666
349	1,30286	1,01249	0,98276
350	1,30417	1,01328	0,9724
351	1,30548	1,01328	0,96411
352	1,3068	1,00956	0,9566
353	1,30812	1,00956	0,96529
354	1,30944	1,00944	0,97365
355	1,31076	1,00944	0,97233
356	1,31209	1,01645	0,97175
357	1,31342	1,01645	0,98221
358	1,31475	1,01519	0,98196
359	1,31609	1,01519	0,9849
360	1,31742	1,00979	0,98132
361	1,31876	1,00979	0,9708
