# Supplementary material for: Water-Dispersible Three-Dimensional LC-Nanoresonators
Source: PLoS One. 2014 Aug 25;9(8):e105474. doi: 10.1371/journal.pone.0105474 (PMC4143276; doi:10.1371/journal.pone.0105474)
Supplement: Table S6 — Figure S1 data. (PDF) [file pone.0105474.s009.pdf]

	A(X)	B(Y)
Long Name	Wavelength	Reflectivity
Units	←m	(a.u.)
Comments		
1	1,17636	0,8341
2	1,17423	0,8343
3	1,1721	0,82976
4	1,16999	0,82743
5	1,16788	0,82918
6	1,16578	0,83146
7	1,16369	0,83078
8	1,1616	0,82945
9	1,15952	0,83026
10	1,15745	0,82861
11	1,15539	0,82778
12	1,15333	0,82638
13	1,15128	0,8254
14	1,14924	0,82831
15	1,14721	0,82844
16	1,14518	0,82598
17	1,14316	0,82575
18	1,14115	0,82508
19	1,13914	0,82379
20	1,13715	0,8229
21	1,13515	0,82083
22	1,13317	0,81966
23	1,13119	0,81858
24	1,12922	0,81888
25	1,12726	0,81923
26	1,1253	0,81929
27	1,12335	0,81747
28	1,12141	0,81544
29	1,11947	0,81399
30	1,11754	0,81254
31	1,11562	0,8132
32	1,1137	0,81134
33	1,11179	0,80666
34	1,10988	0,80544
35	1,10799	0,80517
36	1,1061	0,80415
37	1,10421	0,80281
38	1,10233	0,80043
39	1,10046	0,80104
40	1,0986	0,80026
41	1,09674	0,79666
42	1,09489	0,79629
43	1,09304	0,79711
44	1,0912	0,79529
45	1,08937	0,79363
46	1,08754	0,79291
47	1,08572	0,79255
48	1,0839	0,78987
49	1,08209	0,78756
50	1,08029	0,7873
51	1,07849	0,78607
52	1,0767	0,78481
53	1,07491	0,78388
54	1,07313	0,78265

	A(X)	B(Y)
Long Name	Wavelength	Reflectivity
Units	←m	(a.u.)
Comments		
55	1,07136	0,78237
56	1,06959	0,78209
57	1,06783	0,78083
58	1,06607	0,77901
59	1,06432	0,77752
60	1,06258	0,77526
61	1,06084	0,77399
62	1,05911	0,7737
63	1,05738	0,77287
64	1,05566	0,77111
65	1,05394	0,76906
66	1,05223	0,76818
67	1,05052	0,76702
68	1,04882	0,76586
69	1,04713	0,76491
70	1,04544	0,76365
71	1,04376	0,76308
72	1,04208	0,76246
73	1,04041	0,76156
74	1,03874	0,76096
75	1,03708	0,75955
76	1,03542	0,75836
77	1,03377	0,75781
78	1,03212	0,75754
79	1,03048	0,75652
80	1,02885	0,75599
81	1,02721	0,75609
82	1,02559	0,75563
83	1,02397	0,75538
84	1,02235	0,7554
85	1,02074	0,75418
86	1,01914	0,75317
87	1,01754	0,7536
88	1,01594	0,75371
89	1,01435	0,75295
90	1,01277	0,7525
91	1,01119	0,75257
92	1,00961	0,75251
93	1,00804	0,75263
94	1,00648	0,75199
95	1,00492	0,75156
96	1,00336	0,75213
97	1,00181	0,75247
98	1,00027	0,75196
99	0,99873	0,75249
100	0,99719	0,75286
101	0,99566	0,75349
102	0,99413	0,75326
103	0,99261	0,75305
104	0,99109	0,75328
105	0,98958	0,7533
106	0,98807	0,75392
107	0,98656	0,75455
108	0,98506	0,75468
109	0,98357	0,75472
110	0,98208	0,75461
111	0,98059	0,75537

	A(X)	B(Y)
Long Name	Wavelength	Reflectivity
Units	←m	(a.u.)
Comments		
112	0,97911	0,75661
113	0,97764	0,7571
114	0,97616	0,75746
115	0,9747	0,75772
116	0,97323	0,75848
117	0,97177	0,75869
118	0,97032	0,7586
119	0,96887	0,75941
120	0,96742	0,76023
121	0,96598	0,7594
122	0,96454	0,75896
123	0,96311	0,7606
124	0,96168	0,76166
125	0,96026	0,76197
126	0,95884	0,76235
127	0,95742	0,76337
128	0,95601	0,76408
129	0,9546	0,76336
130	0,9532	0,76314
131	0,9518	0,76349
132	0,9504	0,76398
133	0,94901	0,76501
134	0,94762	0,76614
135	0,94624	0,7668
136	0,94486	0,76724
137	0,94348	0,7671
138	0,94211	0,76804
139	0,94074	0,76829
140	0,93938	0,76786
141	0,93802	0,76908
142	0,93667	0,77059
143	0,93531	0,77026
144	0,93397	0,77038
145	0,93262	0,77103
146	0,93128	0,77172
147	0,92995	0,77236
148	0,92861	0,77239
149	0,92729	0,77284
150	0,92596	0,77359
151	0,92464	0,77309
152	0,92332	0,77179
153	0,92201	0,77279
154	0,9207	0,77387
155	0,91939	0,77385
156	0,91809	0,7735
157	0,91679	0,77476
158	0,9155	0,77732
159	0,91421	0,77716
160	0,91292	0,7754
161	0,91164	0,7751
162	0,91035	0,77571
163	0,90908	0,77699
164	0,90781	0,77714
165	0,90654	0,77759
166	0,90527	0,7783
167	0,90401	0,77938
168	0,90275	0,77704
169	0,90149	0,77623

	A(X)	B(Y)
Long Name	Wavelength	Reflectivity
Units	←m	(a.u.)
Comments		
170	0,90024	0,77846
171	0,89899	0,7792
172	0,89775	0,77937
173	0,8965	0,78008
174	0,89527	0,77871
175	0,89403	0,77747
176	0,8928	0,77915
177	0,89157	0,77866
178	0,89035	0,77808
179	0,88913	0,78013
180	0,88791	0,78122
181	0,88669	0,77982
182	0,88548	0,77824
183	0,88427	0,77781
184	0,88307	0,77885
185	0,88187	0,77888
186	0,88067	0,77731
187	0,87947	0,77684
188	0,87828	0,77551
189	0,87709	0,77581
190	0,87591	0,77826
191	0,87473	0,77769
192	0,87355	0,7775
193	0,87237	0,778
194	0,8712	0,77944
195	0,87003	0,77852
196	0,86886	0,77634
197	0,8677	0,77766
198	0,86654	0,7781
199	0,86538	0,77665
200	0,86423	0,77623
201	0,86308	0,77679
202	0,86193	0,77669
203	0,86079	0,77689
204	0,85965	0,77594
205	0,85851	0,77731
206	0,85737	0,7764
207	0,85624	0,77402
208	0,85511	0,77533
209	0,85398	0,77827
210	0,85286	0,7792
211	0,85174	0,77666
212	0,85062	0,7768
