# Supplementary material for: Water-Dispersible Three-Dimensional LC-Nanoresonators
Source: PLoS One. 2014 Aug 25;9(8):e105474. doi: 10.1371/journal.pone.0105474 (PMC4143276; doi:10.1371/journal.pone.0105474)
Supplement: Table S7 — Figure S2D data. (PDF) [file pone.0105474.s010.pdf]

	A(X)	B(Y)	C(Y)	D(Y)	E(Y)
Long Name	Wavelength	Reflectivity			
Units	←m	(a.u.)			
Comments		1um	1.5um	2um	3um
1	2,6031	0,70667	0,86872	1,00786	0,94778
2	2,59789	0,65153	0,79658	0,96901	0,88275
3	2,59269	0,74361	0,77074	0,861	0,79868
4	2,58752	0,86637	0,75097	0,78335	0,88135
5	2,58236	0,88441	0,74977	0,84542	0,96196
6	2,57723	0,80398	0,88267	0,90864	0,94654
7	2,57211	0,75095	0,92309	0,89068	0,87971
8	2,56702	0,67577	0,80855	0,85156	0,89832
9	2,56195	0,65848	0,79641	0,84154	0,92294
10	2,55689	0,68287	0,86884	0,91183	1,00069
11	2,55186	0,69623	0,82516	0,93631	1,00395
12	2,54685	0,68072	0,75591	0,89449	0,94644
13	2,54185	0,65558	0,84632	0,90575	0,94166
14	2,53688	0,57426	0,83941	0,91706	0,96221
15	2,53192	0,59172	0,69242	0,87865	0,90383
16	2,52699	0,62008	0,70631	0,88438	0,9502
17	2,52207	0,65729	0,77016	0,88756	1,02008
18	2,51718	0,66065	0,72771	0,86543	0,97973
19	2,5123	0,55253	0,65666	0,83222	0,95717
20	2,50744	0,50064	0,59618	0,79014	0,87556
21	2,5026	0,49079	0,55543	0,75408	0,80974
22	2,49778	0,51662	0,57648	0,77765	0,86564
23	2,49297	0,49005	0,61805	0,87609	1,01722
24	2,48819	0,47339	0,6316	0,82163	0,95577
25	2,48342	0,43754	0,65236	0,74538	0,86973
26	2,47867	0,42666	0,60114	0,70901	0,82713
27	2,47394	0,44167	0,6415	0,73954	0,90759
28	2,46923	0,44083	0,64711	0,76774	0,96556
29	2,46453	0,42625	0,55394	0,77709	0,96101
30	2,45986	0,44752	0,53542	0,85977	0,93539
31	2,4552	0,41969	0,51898	0,78979	0,85895
32	2,45056	0,35436	0,55686	0,71637	0,85356
33	2,44593	0,30406	0,59169	0,71178	0,89345
34	2,44133	0,24537	0,53328	0,68771	0,87135
35	2,43674	0,26026	0,43232	0,69978	0,79096
36	2,43217	0,27936	0,40315	0,6751	0,78864
37	2,42761	0,24146	0,40426	0,63448	0,84815
38	2,42308	0,25073	0,42236	0,60286	0,82472
39	2,41855	0,25389	0,42632	0,55195	0,85095
40	2,41405	0,23761	0,40261	0,57131	0,88791
41	2,40956	0,25046	0,37483	0,6116	0,85574
42	2,40509	0,21793	0,3394	0,57538	0,81997
43	2,40064	0,21491	0,29702	0,51403	0,74083
44	2,3962	0,24273	0,30574	0,52016	0,77124
45	2,39178	0,2377	0,30469	0,55473	0,78921
46	2,38738	0,2078	0,25058	0,57674	0,75921
47	2,38299	0,19608	0,23635	0,6222	0,84302
48	2,37862	0,20761	0,28579	0,64915	0,89847
49	2,37426	0,19603	0,28437	0,59533	0,86639
50	2,36992	0,19775	0,24723	0,53697	0,83844
51	2,36559	0,20917	0,21334	0,49316	0,80904
52	2,36128	0,19639	0,24366	0,55012	0,80484
53	2,35699	0,21449	0,2775	0,56818	0,76
54	2,35271	0,27407	0,25091	0,52836	0,75075

	A(X)	B(Y)	C(Y)	D(Y)	E(Y)
Long Name	Wavelength	Reflectivity			
Units	←m	(a.u.)			
Comments		1um	1.5um	2um	3um
55	2,34845	0,26478	0,25383	0,50941	0,76562
56	2,3442	0,22412	0,2931	0,50548	0,80343
57	2,33997	0,20471	0,31853	0,53021	0,8484
58	2,33576	0,20407	0,36228	0,6051	0,85578
59	2,33156	0,22056	0,35905	0,65378	0,87719
60	2,32737	0,22791	0,38638	0,62694	0,88012
61	2,3232	0,24404	0,37906	0,59994	0,87434
62	2,31904	0,26418	0,35982	0,62948	0,85348
63	2,3149	0,26557	0,38261	0,6257	0,87179
64	2,31078	0,29193	0,39853	0,59779	0,86855
65	2,30666	0,29618	0,42659	0,63513	0,84539
66	2,30257	0,31674	0,44972	0,65329	0,83692
67	2,29848	0,33085	0,45745	0,64814	0,84268
68	2,29442	0,34191	0,50265	0,67807	0,88777
69	2,29036	0,34728	0,52912	0,76553	0,90265
70	2,28632	0,3351	0,5333	0,79642	0,89947
71	2,2823	0,32777	0,55298	0,74	0,8982
72	2,27829	0,37237	0,55394	0,73037	0,89219
73	2,27429	0,44079	0,5559	0,79631	0,93688
74	2,27031	0,47346	0,58191	0,83069	0,95818
75	2,26634	0,44609	0,6087	0,77741	0,89873
76	2,26238	0,42233	0,65158	0,74103	0,8417
77	2,25844	0,43867	0,65703	0,77609	0,88491
78	2,25451	0,48299	0,66036	0,82646	0,95762
79	2,2506	0,51534	0,69824	0,83903	0,97894
80	2,2467	0,51484	0,70726	0,83326	0,99909
81	2,24281	0,51578	0,69984	0,82726	0,99169
82	2,23894	0,51283	0,72071	0,87354	1,00458
83	2,23508	0,51593	0,75818	0,92349	0,97578
84	2,23123	0,53326	0,78186	0,9098	0,93875
85	2,2274	0,56874	0,76878	0,90512	0,92309
86	2,22358	0,5876	0,75695	0,90105	0,93255
87	2,21977	0,62068	0,75927	0,88917	0,93986
88	2,21597	0,6319	0,77009	0,91182	0,97241
89	2,21219	0,60794	0,79229	0,95494	1,02578
90	2,20842	0,60387	0,7862	0,96409	1,02996
91	2,20467	0,61986	0,76286	0,91504	0,96968
92	2,20093	0,64727	0,7803	0,88726	0,95924
93	2,1972	0,69002	0,8165	0,90627	0,97969
94	2,19348	0,70009	0,82971	0,95561	0,99007
95	2,18977	0,70081	0,80066	0,92522	0,976
96	2,18608	0,69309	0,79652	0,91433	0,97804
97	2,1824	0,67367	0,7913	0,94124	0,98872
98	2,17873	0,70927	0,79667	0,90741	0,97804
99	2,17508	0,72781	0,85038	0,89557	1,00086
100	2,17143	0,71053	0,84518	0,91333	1,00276
101	2,1678	0,71731	0,8396	0,94437	0,98916
102	2,16418	0,75008	0,86255	0,94138	1,00804
103	2,16058	0,79708	0,86258	0,93142	1,00712
104	2,15698	0,80122	0,87328	0,96597	1,04987
105	2,1534	0,78107	0,88475	0,97145	1,05556
106	2,14983	0,78888	0,89649	0,95856	1,02407
107	2,14627	0,75768	0,93451	0,98621	1,03646
108	2,14272	0,74941	0,88471	0,97152	1,02343
109	2,13918	0,80621	0,83466	0,92738	1,00672
110	2,13566	0,79121	0,86225	0,97446	1,03379
111	2,13215	0,76558	0,85485	0,97578	1,01265

	A(X)	B(Y)	C(Y)	D(Y)	E(Y)
Long Name	Wavelength	Reflectivity			
Units	←m	(a.u.)			
Comments		1um	1.5um	2um	3um
112	2,12865	0,81213	0,86084	0,95142	0,98897
113	2,12516	0,84113	0,92461	1,02138	1,03112
114	2,12168	0,79411	0,93612	1,02737	1,05253
115	2,11821	0,76791	0,87569	0,9647	1,02102
116	2,11476	0,79471	0,83895	0,93486	0,98918
117	2,11131	0,83356	0,85671	0,94807	0,97478
118	2,10788	0,79935	0,8778	0,96157	1,00374
119	2,10446	0,7706	0,85712	0,94683	1,00328
120	2,10105	0,80518	0,81258	0,92277	0,96257
121	2,09765	0,80864	0,835	0,92093	0,9593
122	2,09426	0,79783	0,85764	0,93886	0,97531
123	2,09088	0,81687	0,8599	0,94974	0,98469
124	2,08751	0,86385	0,86691	0,97487	0,9862
125	2,08416	0,88191	0,88224	0,97271	1,0125
126	2,08081	0,88971	0,89575	0,95292	1,00694
127	2,07748	0,89225	0,9254	0,97547	1,03575
128	2,07415	0,90568	0,931	1,01355	1,06454
129	2,07084	0,93504	0,91261	1,01393	1,0611
130	2,06754	0,93042	0,90954	1,00409	1,0903
131	2,06424	0,90094	0,91886	1,01584	1,08286
132	2,06096	0,89946	0,90557	1,00423	1,03666
133	2,05769	0,92605	0,89309	0,99718	1,02181
134	2,05443	0,93138	0,89761	0,99953	1,03864
135	2,05118	0,91687	0,9054	0,99336	1,0417
136	2,04794	0,88981	0,87179	0,98739	1,0347
137	2,04471	0,90122	0,84525	0,98835	1,02638
138	2,04149	0,92879	0,86332	0,98319	1,03169
139	2,03828	0,93108	0,88711	0,98665	1,03587
140	2,03508	0,90984	0,89799	0,99308	1,02998
141	2,03189	0,88145	0,89888	0,99705	1,01794
142	2,02871	0,87589	0,88181	0,97849	1,0003
143	2,02554	0,88329	0,87292	0,95395	0,99234
144	2,02238	0,88722	0,87722	0,96491	1,00016
145	2,01923	0,91714	0,88433	0,95872	0,99069
146	2,01609	0,94739	0,90137	0,97328	1,01368
147	2,01296	0,94277	0,91649	1,01665	1,03777
148	2,00984	0,93313	0,90745	1,00619	1,02712
149	2,00673	0,93387	0,91	0,99018	1,02872
150	2,00362	0,94428	0,92948	1,00767	1,04191
151	2,00053	0,91771	0,93719	1,01069	1,04215
152	1,99745	0,92264	0,90927	0,97098	0,99909
153	1,99438	0,95054	0,90691	0,96741	1,0075
154	1,99131	0,96682	0,91215	0,98217	1,03692
155	1,98826	0,98266	0,90482	0,97619	1,03697
156	1,98521	0,96316	0,91124	1,00689	1,03979
157	1,98218	0,94061	0,89955	1,01074	1,01035
158	1,97915	0,95025	0,90196	0,98927	1,00267
159	1,97614	0,96382	0,91331	0,99902	1,0277
160	1,97313	0,95518	0,9245	0,99865	1,04037
161	1,97013	0,928	0,91552	0,96968	1,02749
162	1,96714	0,92288	0,8903	0,95698	0,99996
163	1,96416	0,94926	0,90213	0,97921	1,01021
164	1,96119	0,9584	0,90762	0,99724	1,02376
165	1,95823	0,96757	0,89601	0,99259	1,00779
166	1,95527	0,9524	0,8939	0,98712	1,01484
167	1,95233	0,92433	0,89106	0,98196	1,02059
168	1,94939	0,92603	0,88089	0,97949	1,01492
169	1,94646	0,94428	0,88833	0,97616	1,01186

	A(X)	B(Y)	C(Y)	D(Y)	E(Y)
Long Name	Wavelength	Reflectivity			
Units	←m	(a.u.)			
Comments		1um	1.5um	2um	3um
170	1,94355	0,93937	0,91535	0,97844	1,00004
171	1,94064	0,95303	0,92489	0,98661	1,00994
172	1,93774	0,9723	0,91919	0,99533	1,02255
173	1,93484	0,96463	0,90787	1,00335	1,01378
174	1,93196	0,95377	0,9006	0,99647	1,01097
175	1,92909	0,96242	0,90416	0,98298	1,01805
176	1,92622	0,95077	0,91883	0,98302	1,02551
177	1,92336	0,93258	0,88991	0,96771	1,00119
178	1,92051	0,9468	0,8694	0,96812	1,00123
179	1,91767	0,95366	0,88273	0,99369	1,02936
180	1,91484	0,95183	0,88418	0,9804	1,0241
181	1,91201	0,94536	0,88849	0,96959	1,03003
182	1,9092	0,95415	0,89749	0,9818	1,03846
183	1,90639	0,97298	0,90553	1,00164	1,03376
184	1,90359	0,95487	0,90941	1,00438	1,03255
185	1,9008	0,94348	0,88854	0,99163	1,0046
186	1,89802	0,95259	0,88517	0,99258	1,0007
187	1,89524	0,94852	0,89482	0,99507	1,01949
188	1,89247	0,94025	0,88739	0,97914	1,0176
189	1,88972	0,93929	0,88067	0,95609	0,98635
190	1,88697	0,94442	0,88736	0,95763	0,97673
191	1,88422	0,94626	0,8996	0,97589	0,9885
192	1,88149	0,95967	0,88263	0,97062	0,98854
193	1,87876	0,96999	0,88183	0,9842	0,99702
194	1,87604	0,95955	0,8952	1,00016	1,01644
195	1,87333	0,95401	0,89913	0,98771	1,02012
196	1,87063	0,95589	0,90155	0,98365	1,00734
197	1,86793	0,96802	0,90337	0,99057	1,00131
198	1,86525	0,96344	0,90508	0,99855	1,0172
199	1,86257	0,96764	0,89942	0,98039	1,01011
200	1,85989	0,99224	0,90955	0,97727	1,00929
201	1,85723	0,99513	0,92282	0,98873	1,02638
202	1,85457	0,98391	0,91805	0,9871	1,02487
203	1,85192	0,9673	0,90705	0,99291	1,02085
204	1,84928	0,97116	0,88301	0,98636	1,00709
205	1,84665	0,98918	0,88058	0,99907	1,02464
206	1,84402	0,98267	0,90329	1,01309	1,03586
207	1,8414	0,95328	0,91335	0,99568	1,02054
208	1,83879	0,94945	0,89721	0,98298	0,9974
209	1,83618	0,96575	0,87745	0,97391	1,00488
210	1,83359	0,97205	0,88968	0,97166	1,02235
211	1,831	0,95471	0,90354	0,98213	1,01863
212	1,82841	0,94281	0,89402	0,96998	0,99246
213	1,82584	0,94157	0,8912	0,96556	0,98192
214	1,82327	0,93683	0,89341	0,9628	0,98744
215	1,82071	0,94469	0,88531	0,96795	0,98358
216	1,81816	0,93968	0,90041	0,97693	0,98858
217	1,81561	0,94799	0,90618	0,96449	0,99097
218	1,81307	0,96202	0,90769	0,96705	0,99238
219	1,81054	0,97338	0,91335	0,9672	0,9942
220	1,80801	0,98962	0,92215	0,98041	1,00618
221	1,8055	0,9957	0,93494	1,01072	1,03127
222	1,80298	0,99138	0,94754	1,01987	1,03814
223	1,80048	0,99291	0,94705	1,01302	1,02281
224	1,79798	0,98647	0,94572	1,01234	1,01829
225	1,79549	0,96689	0,93455	0,99963	1,0183
226	1,79301	0,9617	0,91427	0,98575	1,00326
227	1,79053	0,96875	0,91756	0,99099	1,0096

	A(X)	B(Y)	C(Y)	D(Y)	E(Y)
Long Name	Wavelength	Reflectivity			
Units	←m	(a.u.)			
Comments		1um	1.5um	2um	3um
228	1,78806	0,96891	0,9289	0,98934	1,0279
229	1,7856	0,95966	0,92328	0,98235	1,03131
230	1,78314	0,95135	0,91402	0,98455	1,02144
231	1,78069	0,95898	0,91685	0,98565	1,0126
232	1,77825	0,96425	0,92483	0,97642	1,00826
233	1,77582	0,95939	0,91693	0,97317	1,00386
234	1,77339	0,96709	0,90745	0,99051	0,99663
235	1,77096	0,97474	0,92024	0,99889	1,00227
236	1,76855	0,95203	0,93426	0,99023	1,01192
237	1,76614	0,95273	0,91271	0,97449	1,00089
238	1,76373	0,95677	0,90576	0,98216	1,00756
239	1,76134	0,94983	0,90851	0,98351	0,99448
240	1,75895	0,94819	0,9027	0,9718	0,97991
241	1,75657	0,95254	0,90822	0,97413	0,9856
242	1,75419	0,94965	0,91942	0,98897	1,00666
243	1,75182	0,95876	0,91754	0,98524	1,01427
244	1,74945	0,97406	0,91772	0,9764	1,00443
245	1,7471	0,98792	0,93286	0,98571	1,00016
246	1,74474	0,97738	0,94125	0,99894	1,01132
247	1,7424	0,95601	0,93794	1,00159	1,01662
248	1,74006	0,94559	0,93138	0,98243	1,01344
249	1,73773	0,95252	0,92512	0,9693	1,01352
250	1,7354	0,94681	0,92911	0,97907	1,01348
251	1,73308	0,94339	0,9133	0,97848	0,99242
252	1,73077	0,94289	0,90531	0,9742	0,97877
253	1,72846	0,94393	0,91616	0,98029	0,98812
254	1,72616	0,94973	0,91472	0,98175	0,99799
255	1,72386	0,95052	0,90492	0,98206	1,0025
256	1,72157	0,95067	0,89877	0,98482	1,00132
257	1,71929	0,94363	0,89836	0,97117	0,99814
258	1,71701	0,93463	0,90059	0,95142	0,98939
259	1,71474	0,93375	0,91023	0,9506	0,98865
260	1,71248	0,93239	0,90968	0,96866	0,99651
261	1,71022	0,92791	0,89838	0,97177	0,9922
262	1,70796	0,92803	0,88642	0,95929	0,9772
263	1,70572	0,94237	0,88815	0,95457	0,97775
264	1,70348	0,94128	0,90222	0,96389	0,99272
265	1,70124	0,93212	0,90377	0,96873	0,99118
266	1,69901	0,94178	0,89968	0,95474	0,97465
267	1,69679	0,95896	0,91021	0,96448	0,98673
268	1,69457	0,95268	0,92327	0,98038	1,01106
269	1,69236	0,94931	0,92112	0,96687	0,99986
270	1,69015	0,96191	0,91029	0,96372	0,98782
271	1,68795	0,96496	0,90698	0,97723	0,99371
272	1,68575	0,96536	0,90923	0,98038	0,99114
273	1,68357	0,9556	0,91959	0,97687	0,99627
274	1,68138	0,93855	0,92637	0,98121	1,00512
275	1,6792	0,93578	0,91092	0,97954	1,00052
276	1,67703	0,93508	0,89922	0,96686	0,98867
277	1,67486	0,92679	0,89287	0,95838	0,97294
278	1,6727	0,92969	0,88093	0,95277	0,96684
279	1,67055	0,93174	0,88521	0,95393	0,9809
280	1,6684	0,92679	0,8901	0,95848	0,98425
281	1,66625	--	0,8827	0,96344	0,97841
