# Supplementary material for: Water-Dispersible Three-Dimensional LC-Nanoresonators
Source: PLoS One. 2014 Aug 25;9(8):e105474. doi: 10.1371/journal.pone.0105474 (PMC4143276; doi:10.1371/journal.pone.0105474)
Supplement: Text S1 — Contrast of randomly oriented nanoresonators. (DOCX) [file pone.0105474.s011.docx]

**Text S1: Contrast of randomly oriented nanoresonators**

The reflectivity spectrum (Figure S1) of pulled off nanoresonators randomly oriented was measured with a Fourier-trasform (FTIR) spectrometer, using a commercial insert for fixed 10° incidence angle (Pyke 10 Spec), a white lamp as source and a Si detector.

The resonance is blue-shifted to around 1 μm (as expected) with respect to the nanoresonators discussed in the article, because the size of the present ones was smaller (100*200 nm) and the dielectric layer was thicker (100 nm). From a simple nanocircuit model, we can justify this result, observing that the ‘capacitance’ of the nanoresonator was decreased, and this causes an increase of the resonance frequency. The measurement of pulled off nanoresonators gives us an idea of the expected coupling when dispersed in liquid solution, where they are also randomly oriented. In this case of pulled off nanoresonators on a GaAs substrate, we found a contrast of 3/4 %, and so we have to expect a lower one in solution for the chemical binding necessary to suspend the nanoresonators (that acts as an external resistance) and the fully random orientation in three dimensions.
