# Supplementary material for: Water-Dispersible Three-Dimensional LC-Nanoresonators
Source: PLoS One. 2014 Aug 25;9(8):e105474. doi: 10.1371/journal.pone.0105474 (PMC4143276; doi:10.1371/journal.pone.0105474)
Supplement: Text S2 — Easy tailoring of shape, size and materials. (DOCX) [file pone.0105474.s012.docx]

Text S2: Easy tailoring of shape, size and materials

The LC behaviour of a bone shape results more obvious as the role of capacitances and inductance is readily apparent. In Figure S2A such design of LC nanoresonators is shown, realized with the same top-down approach of the simpler brick shape described in the article.

In the extremities there are two square prisms joined by another rectangular one. This shape reminds a ‘bone’ whose sizes are: L= 155nm, l, w= 125 nm, t= 50 nm, h= 100 nm. The nanoresonator is made of gold (top layer), gallium arsenide (central layer) and gold (bottom layer). Only the first two layers are defined. Figure S2B displays the simulations of the electric (z-component) and magnetic (norm) fields in the plane (x,z) and (x,y). The capacitive element is due to the electric field that is concentrated in the extremities (plates of the capacitors) while the inductive behaviour is generated by the magnetic field around the central part of the nanoresonator (the ‘wire’). This clearly demonstrates the tailorable versatility of our nanoresonators with respect to common plasmonic nanoparticles. A SEM image of LC nanoresonators is shown in Figure S2C and their characterization is reported in Figure S2D. For this Reflectivity spectrum we used the M-polarization (magnetic field perpendicular to the ‘wire’). It is important to note that the size of the nanoresonator produces a resonance at longer wavelength (2.4 μm) (as expected from the simulations). It is an example of how our nanofabrication process permits a huge choice of sizes and shapes, responding in various spectral regions and for different applications, simply changing the CAD of the EBL process. The reflectivity spectrum for different distances among nanoresonators (1, 1.5, 2, 3 μm) demostrates that the position of the resonance depends on the single nanoresonators (size and shape) and not on the array structure, even if the signal decreases with the distance of the nanostructures due to the smaller number of excited nanoresonators.
