# Supplementary material for: Water-Dispersible Three-Dimensional LC-Nanoresonators
Source: PLoS One. 2014 Aug 25;9(8):e105474. doi: 10.1371/journal.pone.0105474 (PMC4143276; doi:10.1371/journal.pone.0105474)
Supplement: Text S3 — Very large tunability. (DOCX) [file pone.0105474.s013.docx]

**Text S3: Very large tunability**

With our nanofabbrication process we are able to nanofabricate the nanoresonators with very large tunability of the resonance. For example in Figure S3 we fabricated a LC nanoresonator (with circular shape and Au,GaAs,Au materials) for the Terahertz range, simply scaling the sizes from nanoscale to microscale. This nanoresonator is very similar to the one discussed in Walther et al. (see article, reference [21]).
